# Three members of *Medicago truncatula* ST family are ubiquitous during development and modulated by nutritional status (MtST1) and dehydration (MtST2 and MtST3)

**DOI:** 10.1186/s12870-017-1061-z

**Published:** 2017-07-10

**Authors:** Lucía Albornos, Ignacio Martín, Emilia Labrador, Berta Dopico

**Affiliations:** Departamento de Botánica y Fisiología Vegetal. Instituto Hispano-Luso de Investigaciones Agrarias (CIALE), University of Salamanca. C/ Licenciado Méndez Nieto s/n, Campus Miguel de Unamuno, 37007 Salamanca, Spain

**Keywords:** Abiotic stress, *cis*-acting regulatory elements, Development, DUF2775, *Medicago truncatula*, ST protein

## Abstract

**Background:**

ShooT specific/Specific Tissue (ST) belong to a protein family of unknown function characterized by the DUF2775 domain and produced in specific taxonomic plant families, mainly Fabaceae and Asteraceae, with the *Medicago truncatula* ST family being the largest. The putative roles proposed for this family are cell elongation, biotic interactions, abiotic stress and N reserve. The aim of this work was to go deeper into the role of three *M. truncatula* ST proteins, namely ST1, ST2 and ST3. Our starting hypothesis was that each member of the family could perform a specific role, and hence, each *ST* gene would be subjected to a different type of regulation.

**Results:**

The search for *cis*-acting regulatory elements (CREs) in silico in *pST1, pST2* and *pST3* promoters showed prevalence of tissue/organ specific motifs, especially root- and seed-specific ones. Light, hormone, biotic and abiotic related motifs were also present. None of these *pSTs* showed the same combination of CREs, or presented the same activity pattern. In general, *pST* activity was associated with the vascular cylinder, mainly in roots. Promoter activation was highly specific and dissimilar during reproductive development. The *ST1*, *ST2* and *ST3* transcripts accumulated in most of the organs and developmental stages analysed - decreasing with age - and expression was higher in the roots than in the aerial parts and more abundant in light-grown plants. The effect of the different treatments on transcript accumulation indicated that *ST1* behaved differently from *ST2* and *ST3*, mainly in response to several hormones and dehydration treatments (NaCl or mannitol), upon which *ST1* transcript levels decreased and *ST2* and *ST3* levels increased. Finally, the ST1 protein was located in the cell wall whereas ST2 and ST3 were present both in the cytoplasm and in the cell wall.

**Conclusions:**

The ST proteins studied are ubiquitous proteins that could perform distinct/complementary roles in plant biology as they are encoded by differentially regulated genes. Based on these differences we have established two functional groups among the three STs. ST1 would participate in processes affected by nutritional status, while ST2 and ST3 seem to act when plants are challenged with abiotic stresses related to water stress and in physiologically controlled desiccation processes such as the seed maturation.

**Electronic supplementary material:**

The online version of this article (doi:10.1186/s12870-017-1061-z) contains supplementary material, which is available to authorized users.

## Background

STs (ShooT specific, as defined for the first time by De Vries et al. [1], and also referred to as Specific Tissue in the databases) belong to a protein family of unknown function found in specific taxonomic plant families, mainly Fabaceae and Asteraceae, but not in Brassicaceae. Members of this family were first described as cDNA clones in garden pea and chickpea etiolated epicotyls [1–4], and a comprehensive in silico characterization of the ST family was performed by Albornos et al. [5].

ST proteins are encoded by multigenic families, where the largest family belongs to *Medicago truncatula* and is comprised of 6 members. The main feature of STs is the presence of at least one DUF2775 domain whose function is unknown. These proteins have a signal peptide, an N-terminal region with conserved characteristics and several tandems repeats of 25/26 amino acids in variable number [5]. However, ST proteins cannot be associated with any of the previously described members of the category II tandem repeat proteins proposed by Katti et al. [6]. All repeats share a hexapeptide followed by four partially conserved amino acids and a fully conserved tyrosine at position 11 [5]. The repeats are highly conserved within the same ST protein, and the variations among STs are enough to separate them into three groups according to their putative glycosylation pattern (see [5] for a more detailed description of the proteins).

It is difficult to assign a subcellular location to ST proteins based on in silico predictions even though they contain a signal peptide, indicating their entrance into the secretory pathway, because they lack any other subcellular targeting signals. Recent results from our group point to a dual location of the *Cicer arietinum* ST1 protein, which is both intra- and extracellular, but the location of other ST proteins still needs to be confirmed [7].

An expressed sequence tag (EST) profile was determined for the ST family after the analysis of publicly available cDNA libraries in which *ST* clones were identified [5]. Within the plant, 49% of the sequences came from root and/or radicle libraries, and to a lesser extent from libraries made out of mRNA from seeds (9%), leaves (7%) and stems, epicotyls and hypocotyls (6%). Numerous sequences came from libraries originating from plants grown under biotic interactions (21%) or abiotic stresses (15%). Additionally, the *M. truncatula* gene expression atlas (MtGEA), also publicly available, confirmed the predominance of transcript accumulation in roots [8, 9].

Several putative roles for ST proteins in plant physiology can be inferred from the information obtained so far in vivo and in silico, which could include these proteins being related to biotic interactions, since their possible involvement in symbiosis has been repeatedly reported [10–14], and to abiotic stress [3, 15]. Also, different members of the ST family have been reported to be related to developmental processes such as early fruit morphogenesis [16–19], cell elongation [2, 3] or germination [20], and a putative role as vegetative N storage has also been proposed [2, 7].

To date, several studies regarding the ST family have been carried out in chickpea [3, 5, 7, 15], but although its genome has recently been sequenced [21] it still remains a species difficult to transform. In contrast, *M. truncatula* is a well-established model legume with a sequenced genome [22], and there are numerous laboratory protocols and tools available that facilitate its use. Thus, we decided to study the *ST* genes in this species, which is also the biggest ST family and comprised of six members. Barrel medic *ST* genes, namely *MtST1* (Medtr4g069810), *MtST2* (Medtr3g116440), *MtST3* (Medtr3g116430), *MtST4* (Medtr3g034640), *MtST5* (Medtr3g034610) and *MtST6* (Medtr3g107810) encode proteins with a similar structure. *MtST1* is located on chromosome 4 and encodes a type I ST, while the other five genes are located on chromosome 3 and encode proteins belonging to type II, as described in Albornos et al. [5]. After taking into account that the EST profile and the MtGEA microarrays showed that *MtST* transcripts might be differentially distributed throughout the plant, and considering that ST proteins from different species have been associated with diverse functions, we set out to determine if members of the *M. truncatula* ST family played different or complementary roles in plant physiology, and if their gene expression was differentially regulated.

The aim of this work was to unravel the possible functions of 3 of the 6 ST proteins of *M. truncatula*, namely MtST1, MtST2 and MtST3 (ST1, ST2 and ST3 in this manuscript), by analysing the promoter sequences of these 3 *ST* genes in silico, by establishing promoter activity in *Arabidopsis thaliana* transgenic plants, by studying their transcript accumulation throughout plant development, and in response to different growth conditions, and by determining the subcellular locations of these ST protein.

## Results

### The *ST1* ORF was mis-annotated in the databases

To perform this work, we cloned the promoters and open reading frames (ORFs) of the *ST1*, *ST2* and *ST3* genes using the sequences annotated in Phytozome database [22]. The analyses of the annotated sequences confirmed that the gene structure of *ST1*, *ST2* and *ST3* was characteristic of the *ST* gene family [5] containing introns of 826, 2361 and 693 bp in length, respectively. Furthermore, the analysis of the *ST3* gene sequence, as well as its cloned mRNA, indicated that a putative mutation introduces a premature stop codon in the genome sequence, which explains why ST3 C-terminal end is not canonical [5].

Therefore, the cloned promoters and ORFs of the three genes allowed us to compare their sequences with those found in the databases; only the cloned *ST1* ORF showed differences between its sequence and the annotated sequence. After analysing several clones, two differences were confirmed: the first one is the presence of an extra codon for Lys in the annotated ORF, probably because of a wrong in silico interpretation of intron removal, and the second one is a discrepancy with the number of tandem repeats; 11 were present in the database sequence and 13 in the cloned ORF.

The three encoded acidic proteins had the canonical ST protein structure: a signal peptide (21 or 22 aa); a non-repeated sequence (from 43 to 74 aa) and a 26 aa sequence repeated in tandem [5]. ST1, ST2 and ST3 displayed 13, 10 and 14 tandem repeats, respectively, which gave rise to proteins of different length and molecular masses. ST1 and ST3 had 430 aa with a calculated MW of 48.6 kDa and ST2 had 359 aa and a MW of 40.7 kDa.

### Several cis-acting regulatory elements were present in *ST1*, *ST2* and *ST3* promoter sequences

In silico analysis of promoters has become an important source of information to study the transcriptional regulation of genes. Core promoters can be TATA or TATA-less depending on the elements they have to initiate transcription. The TSSP software predicted the transcription start site (TSS) (−58, −47 and −69 for *pST1*, *pST2* and *pST3*, respectively), and the presence of a TATA box (−92, −81 and −102 for *pST1*, *pST2* and *pST3*, respectively) in the three promoters studied, with a linear discriminant function over 0.02 that validates the predictions.

An ever-increasing number of cis-acting regulatory elements (CREs) are being collected in the databases. However, their presence within a given promoter of interest and their association with a putative activation profile requires in vivo analysis.

**Fig. 1:**
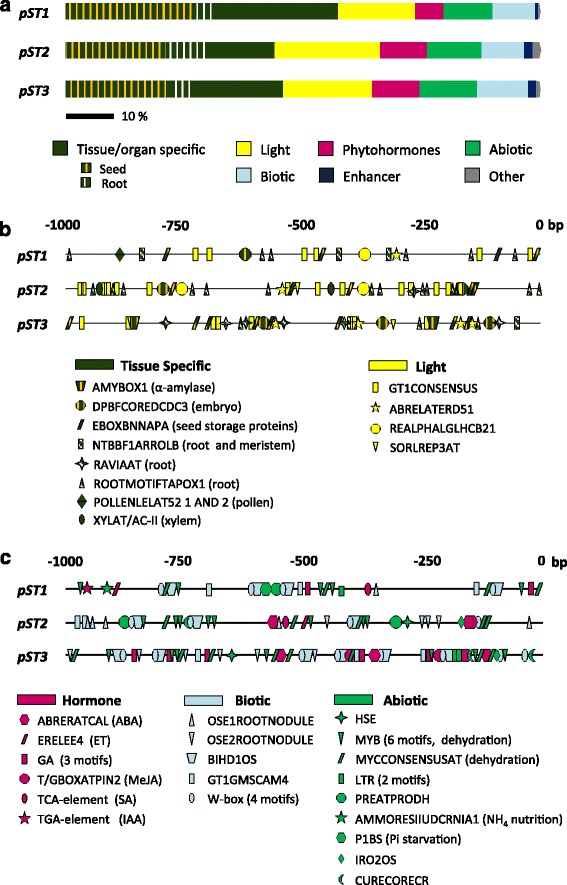
CREs present in *pST1*, *pST2* and *pST3* according to in silico predictions using PLACE and PlantCARE. **a** Percentage of CREs belonging to the different categories established in this work. **b** Position of CREs in the tissue specific and light categories*.* **c** Position of CREs in the hormone, biotic and abiotic categories. Highly abundant CREs (ARRIAT, CAATBOX, GATABOX, GTGANTG10, DOFCOREZM) were not represented in order to simplify the figure. bp: base pairs upstream ATG

Examination of the first 1000 bp in *ST* promoters (*pST*s) using the PLACE and PlantCARE databases identified 113 different CREs (614 motifs). *pST1* had the least number of motifs (166) and *pST2* and *pST3* had 225 and 223, respectively (Additional file 1). The CREs were classified into 7 categories, according to the functional information provided by the databases (Fig. 1a). These categories were: 1) CREs controlling specific activities in organs or tissues; 2) related to light regulation; 3) hormone response; 4) establishment of biotic interactions; 5) abiotic stress response; 6) enhancers; and 7) unclassified (other) (Additional files 1 and 2). Each of the three *pSTs* had its own specific combination of CREs (Fig. 1b, c; Additional file 2).

The elements within the tissue/organ specific category, which represented at least 44% of the motifs found in each of the *pST*s, were those associated with the meristem, root, mesophyll, flower and seed subcategories, and were present in all three *pST*s. In contrast, the CREs found in xylem were only present in *pST2*, CREs in guard cells were only present in *pST1* and *pST3*, and CREs in fruit were only present in *pST1* (Fig. 1b; Additional file 1). The most widespread motif in the meristem subcategory, NTBBF1ARROLB, which is associated with apical meristems and specifically with vascular tissues and auxin induction, was absent in *pST2* (Fig. 1b; Additional file 2). The CREs found in the root subcategory were especially frequent in *pST2* (9%)*.* However, the ROOTMOTIFTAPOX1 element, specific to the roots of adult plants, was the only CRE found in all of the *pST*s, and was the most abundant in *pST1* and *pST2*. RAV1AAT, related to gene expression in rosette leaves, was most abundant in *pST3* (Additional file 2).

Known CREs associated with reproductive development were also found in the *pST*s. Flower-specific elements were well represented (6–8%) mostly those associated with pollen expression (Additional file 2). The motifs GTGANTG10 and POLLEN1LELAT52 were found in all three *pSTs* however, POLLEN2LELAT52, the co-dependent element of POLLEN1LELAT52, was only present in *pST1*. The high number of seed-specific CREs is also noteworthy, being more abundant in *pST1* (27%) than in *pST2* and *pST3* (21% and 22%, respectively). Seed motifs were divided into monocot or dicot subcategories, depending on their origin (Additional file 1). *pST1* and *pST3* presented nearly the same number of motifs of both subcategories, while *pST2* had fewer of the elements found in monocot seeds (8% vs. 13%). A total of 20 different seed-related CREs, 11 of them found in the promoters of genes encoding seed storage proteins, was also present in *pST1*, *pST2* and *pST3* (Fig. 1b; Additional file 2).

Numerous light-associated CREs (ranging from 16 to 22%) were found in the *pSTs* (Additional file 1). Three different subcategories were considered: light-regulated elements (LRE), with 22 motifs in the three *pST*s; circadian elements (1 motif found in *pST3*); and phytochrome elements (2 motifs absent in *pST3*) (Fig. 1b; Additional file 1). Among the LREs, the most represented motifs were the 4 bp GATABOX (32 times), conserved in the promoters of genes encoding chlorophyll a/b binding proteins, and GT1CONSENSUS (20 times), a GT-1 binding site mainly related to light regulation, prevalent in *pST2* (Additional file 2). These CREs, together with IBOX and ABRELATERD, were the only ones found in all of the *pST*s (Additional file 2). Apart from ABRELATERD, another two motifs associated with gene expression in darkness, REALPHALGLHCB21 and SORLREP3AT, were also found (Fig. 1b; Additional file 2).

The category of CREs involved in the hormonal regulation of gene expression represented approximately 10% of the elements found in *pST2* and *pST3*, but only 6% in *pST1* (Additional file 1). CK elements, mainly ARR1AT, were the most abundant and the only ones found in every *pST*, reaching 7% in *pST2* (Additional file 1). ABA motifs, such as ABRERATCAL, appeared in *pST2* and *pST3* but did not appear in *pST1*. The unique IAA element was exclusively found in *pST1*, while GA elements were absent in *pST2*. Promoters containing ET- and SA-related CREs (*pST1* and *pST2*) lacked JA specific elements (*pST3*) and vice versa (Fig. 1c; Additional files 1 and 2).

The *pST*s also contained elements related to biotic interactions, including symbiosis and pathogenesis, that ranged from 9% (*pST1* and *pST2*) to 11% (*pST3*) (Additional file 1). There were two motifs related to symbiosis known to drive gene expression in the infected cells of root nodules in different species including *M. truncatula*: OSE1ROOTNODULE, which was absent in *pST3*, and OSE2ROOTNODULE present in all three *pST*s (Fig. 1c; Additional file 1). The pathogenesis subcategory contained 9 different CREs that appeared 39 times overall (Additional file 1). The most represented were BIHD1OS (13 matches), associated with disease, and GT1GMSCAM4 (8 matches), related to pathogen attack and salinity (Fig. 1c; Additional file 2). The presence of 4 different W-box binding motifs of the WRKY transcription factors (TF) (14 matches) is also worth noting (Additional file 2).

Elements associated with abiotic stress represented 10%, 11% and 12% of the motifs in *pST1*, *pST2* and *pST3*, respectively (Additional file 1). Within this category we established five subcategories. A small number of CREs related to anoxia (absent in *pST3*) and heat shock (absent in *pST1*) were found. All three *pST*s contained elements associated with dehydration, low temperature and nutrition subcategories. Dehydration motifs, including water stress and drought tolerance, were the most abundant CREs (Additional file 1). Six of the 9 drought-response elements were MYB transcription factor binding sites (20 matches altogether) and 1 was the MYC transcription factor binding site MYCCONSESUSAT, the most represented element in the abiotic category (17 matches) (Fig. 1c; Additional file 2). In the nutrition subcategory, the sulphur deprivation element (SURECOREATSULTR11) was found in all three *pST*s, while the ammonium-response (AMMORESIIUDCRNIA1) and phosphate-starvation (P1BS) elements were unique to *pST1*. In contrast, iron starvation (IRO2OS) and copper responsive elements (CURECORECR), present in *pST2* and *pST3*, were absent in *pST1* (Additional file 2).

Within the enhancer regions category, we distinguished between general enhancers, such as 2 TA-rich enhancer motifs in *pST3,* and specific enhancers that increase transcription in specific genes or tissues, such as the Skn-1 motif, related to endosperm, in *pST1* and *pST2* (Additional files 1 and 2). Finally, in the category of others, we included elements involved in cell cycle and gene expression in plastids, which represented less than 2% of CREs found in all three *pST*s (Additional files 1 and 2).

### Activity of *pST1*, *pST2* and *pST3* in *Arabidopsis thaliana* was high in the root vascular cylinder and showed maximum differences during reproductive development

**Fig. 2:**
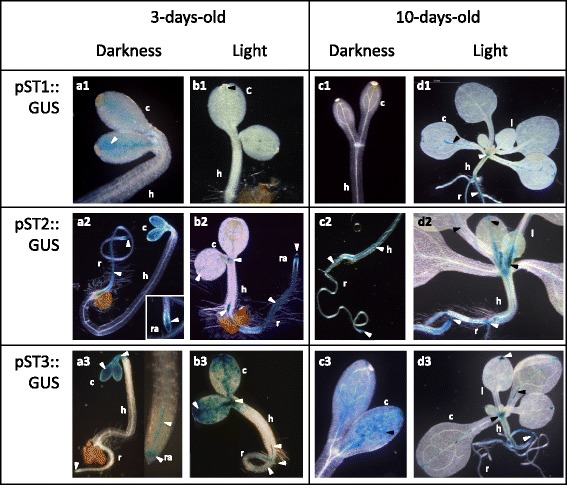
Activity of *Medicago truncatula pST1*, *pST2* and *pST3* in *Arabidopsis thaliana* seedlings. Histochemical GUS staining in transgenic pST::GUS seedlings at 3- (**a**, **b**) and 10-d-old (**c**, **d**) grown in *darkness* (a, c) or *light* (b, d). **Numbers** refer to the corresponding *ST* gene. c, cotyledon; h, hypocotyl; l, leaf; r, root; ra, root apex. The *blue colour* and the *arrows* indicate the zones with GUS activity driven by *pST*

**Fig. 3:**
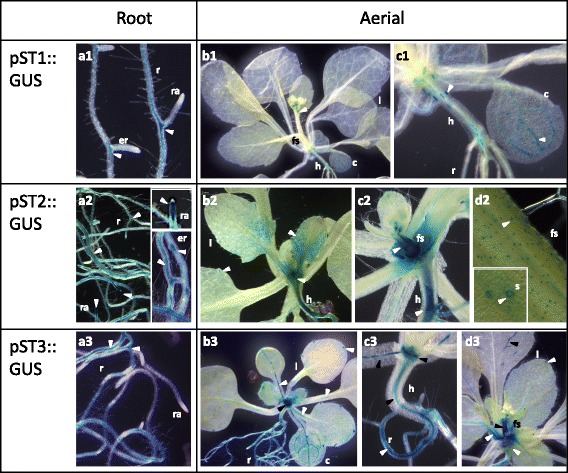
Activity of *Medicago truncatula pST1*, *pST2* and *pST3* in vegetative organs of *Arabidopsis thaliana* plants*.* Histochemical GUS staining in vegetative organs (**a**, roots; **b**-**d**, aerial parts) of 10- to 24-d-old pST::GUS transgenic plants. Numbers refer to the corresponding *ST* gene. c, cotyledon; er, emerging root; fs, floral stem; h, hypocotyl; l, leaf; r, root; ra, root apex; s, stoma. The *blue colour* and the *arrows *indicate the zones with GUS activity driven by *pST*

**Fig. 4:**
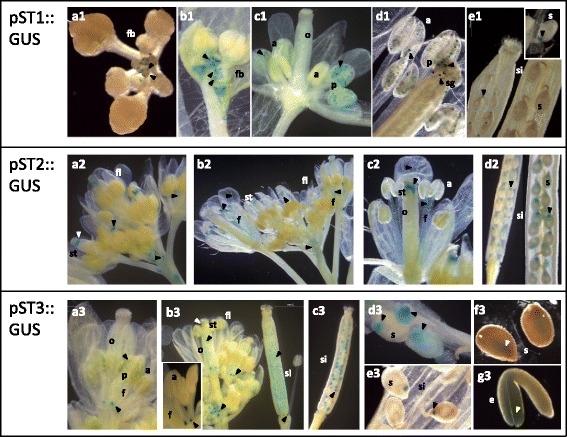
Activity of *Medicago truncatula pST1*, *pST2* and *pST3* in reproductive organs of *Arabidopsis thaliana.* Histochemical GUS staining during reproductive development of arabidopsis pST::GUS plants (**a** to **g** sorted according to developmental stage). Numbers refer to the corresponding *ST* gene. a, anther; e, embryo; f, filament; fb, floral bud; fl, flower; o, ovary; p, pollen; s, seed; sg, stigma; si, silique; st, style. The *blue colour* and the *arrows* indicate the zones with GUS activity driven by *pST*

Arabidopsis transgenic plants with a *ST* gene promoter driving the expression of the *GUS* reporter gene (pST::GUS) were generated to determine the activity of these promoters. The wild-type plants used as controls did not show GUS activity; therefore, no images have been included. Two *pST3* constructs of different lengths were made: *pST3*·F1 refers to the longest construct consisting of 2390 bp, and *pST3*·F2 to the shortest one with 1073 bp. Both constructs displayed the same activity pattern, with *pST3*·F1 activity higher than *pST3*·F2 in vegetative organs. All of the images shown in Figs. 2, 3, and 4 came from the pST3·F1::GUS transgenic plants.

The analysis of the activity of the *pST*s was carried out in 3- (Fig. 2a, b) and 10-d-old (Fig. 2c, d) dark-grown (etiolated) (Fig. 2a, c) and light-grown (green) (Fig. 2b, d) seedlings. In general, GUS activity was found mainly in the cotyledons and root vascular cylinder, and activity in the cotyledons was higher in the etiolated and youngest plants (Fig. 2). All three promoters showed activity in the cotyledons of 3-d-old etiolated and green seedlings (Fig. 2a, b); activity was the faintest in the green seedlings (Fig. 2b). At 3-d-old, *pST2* and *pST3* were also active in roots, both in the apex and in the vascular cylinder, although at different intensities (Fig. 2a2, a3, b2, b3). In dark-grown seedlings *pST3*, but not *pST2*, was also active at the hook level (Fig. 2a3). The activity of *pST2* and *pST3* observed in the shoot apex and in the root-hypocotyl junction in light-grown seedlings (Fig. 2b2, b3) was quite intense.

Almost no activity, except for *pST3*, was found in the cotyledons of 10-d-old etiolated seedlings (Fig. 2c). In these plants, only *pST2* remained slightly active in the roots, and its activity extended towards the hypocotyl vascular cylinder (Fig. 2c2). The three promoters were active in 10-d-old light-grown pST::GUS plantlets (Fig. 2d), and their activation pattern was maintained in the vascular cylinder, either in leaves, hypocotyl and/or radicles, as vegetative growth progressed (Fig. 3) regardless of the plants’ age. In most cases, the roots (Fig. 2d, 3a) were more stained than the above ground tissues (Fig. 3b–d), where blue staining was observed mainly in the leaf veins and hypocotyl. However, differences were observed in the activity pattern of each *ST* promoter.

The analysis of the promoter activities in roots from 10 to 24-d-old transgenic plants (Fig. 3a) showed that *pST1* and *pST3* were inactive in the root apex (Fig. 3a1, a3), unlike *pST2* that was active both in primary and secondary root apical areas (Fig. 3a2). Although the activity of *pST1* was discontinuous, blue staining was mainly located in the vascular cylinder, especially where the econdary roots emerged (Fig. 3a1). The most active promoter in roots was *pST2*, showing blue staining throughout the central cylinder, both in primary and secondary roots. The darkest staining was observed in the insertion zone of secondary roots, where activity was initiated as soon as a root started to develop (Fig. 3a2). By contrast, pST3::GUS plants showed higher GUS activity in the basal area of their primary root, being fainter or absent in the rest of the root and with no activity in the secondary roots (Fig. 3a3, c3).

Regarding the aerial organs, *pST2* and *pST3* were not very active, and the staining was restricted to hydathodes in the leaves and veins of the cotyledons (Fig. 3b2, b3, d3), being almost undetectable in the pST1::GUS transformants (Fig. 3b1, c1). pST2::GUS and pST3::GUS plants showed blue colour in the lamina of the youngest leaves, mainly in the proximal part of the organ, (Fig. 3b2, b3) but only *pST3* was active in the vascular bundle of leaf petioles (Fig. 3b3, c3). Also, two different *pST* activity patterns were observed (Fig. 3c) in the hypocotyl. While *pST1* was active only in some individual cells in the epidermis and vascular cylinder (Fig. 3c1), *pST2* and *pST3* showed a highly continuous GUS activity, but only in the central cylinder (Fig. 3c2, c3). *pST2* and *pST3* were active in the centre of the rosette (Fig. 3c2, c3, d3), most probably where new leaves start to develop or when the flower stem starts to emerge. In fact, both promoters were active in the flowering stem, although with different patterns; *pST2* acted in the veins (Fig. 3c2) and, once developed, in guard cells (Fig. 3d2 and detail in the square). *pST3* was seen throughout the stem, but it was difficult to determine its precise activity (Fig. 3d3).

The activity of the three *pST*s differed greatly during flower and silique formation (Fig. 4), confirming their specificity during reproductive development. Fig. 4 shows the structures with GUS activity sorted according to their developmental stage from the beginning of flower formation to the mature silique.

The activity of *pST1* along flower development was restricted to the androecium (Fig. 4, a1-d1). High *pST1* activity was detected in 4 specific points at the base of flower bud, coinciding with the origin of the four long stamens (medial) (Fig. 4a1, b1). *pST1* was active during pollen formation, from medium stages to maturity (Fig. 4c1, d1). In fact, the pollen of long stamens stained an intense colour of blue earlier than the pollen from the short stamens (lateral) (Fig. 4c1). During seed and fruit development, the GUS activity driven by *pST1*, was only observed in the joint between funiculus and seed at early/medium stages of fruit development (Fig. 4e1 and detail).

*pST2* was style specific in the beginning of flower growth, and this activity continued along gynoecium development (Fig. 4a2-c2). However, later on in development, blue staining was also observed in the stamen filament, close to the anthers and all along its vascular tissue (Fig. 4a2, b2, c2). A faint GUS activity was also observed in petal and pedicel vasculature, as well as in the pedicel insertion point (Fig. 4a2, b2, c2). The activity of *pST2* was transiently up regulated in the chalazal endosperm tissue at a specific seed developmental stage (Fig. 4d2).

Unlike *pST1* and *pST2*, *pST3* was related mainly to female gametophyte development. In addition, *pST3* was active at the base of stamen filaments along their development (Fig. 4a3, b3), in several pollen grains (Fig. 4a3), and in the style at initial flower growth (Fig. 4b3). It was also possible to detect blue staining in the female gametophyte before fertilization (Fig. 4b3), and the activity of the promoter was maintained but decreased throughout seed development (Fig. 4b3-f3). At the beginning of seed development dark blue staining was observed in the whole seed, within the endosperm and embryo (Fig. 4d3). Later, the activity decreased and finally was restricted to the chalazal endosperm, the same that was observed for *pST2* (Fig. 4d3, e3). Finally, faint GUS activity remained in embryonic cotyledons of pST3::GUS plants (Fig. 4f3, g3).

### *ST1*, *ST2* and *ST3* transcript accumulation was ubiquitous during plant development and specific under different growth conditions

**Fig. 5:**
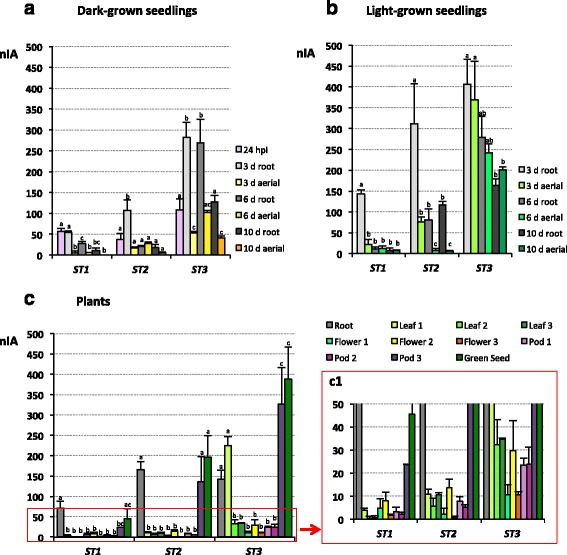
sqRT-PCR of *Medicago truncatula ST1*, *ST2* and *ST3* transcripts along development. **a** Transcript accumulation in 24 hpi seeds and in 3-, 6- and 10-d-old dark-grown seedlings (roots and aerial parts). **b** Transcript accumulation in 3-, 6- and 10-d-old light-grown seedlings (roots and aerial parts). **c** Transcript accumulation in several organs of adult plants: root from 30-d-old plants; leaves, flowers and pods in 3 developmental stages; and green seed. The different development stages of leaves were: Leaf 1, small, closed folioles without spot; Leaf 2, fully expanded leaves, folioles with spot smaller than 1 cm; and Leaf 3, fully expanded leaves, folioles with spot bigger than 1 cm. The different developmental stages of flowers were: Flower 1, *green* immature with sepals covering the organs; Flower 2, closed *yellow* flower two days before anthesis; and Flower 3, at anthesis. The different developmental stage of pods were: Pod 1, early pod with a complete spiral; Pod 2, pod with 5 complete spirals and initial spines with embryo at globular state; and Pod 3, pod with 6 complete spirals and mature spines with embryo at heart state. *Green* seeds are seeds collected from 24 to 26 d after pollination. **c1** Detail of bars between 0 and 50 units showed in c (*red square*). Transcript accumulation was measured relative to ubiquitin as indicated in the Methods section. Scale units express normalized and integrated absorbance (nIA). More detailed description of plant material is included in Additional file 3. Statistical analyses were performed separately for each *ST* gene as stated in the Methods section. The different *letters* on the tops of the histograms indicate a statistically significant difference of *p* < 0.05

Analysis of *M. truncatula ST1*, *ST2* and *ST3* transcript accumulation during development was performed in seeds 24 h post imbibition (hpi) throughout the development of dark- and light-grown seedlings and in adult green plants (Fig. 5). Photographs of *M. truncatula* at the stages studied are showed in Additional file 3 and the agarose gel electrophoresis of sqRT-PCR amplicons in Additional file 4. Only those changes statistically significant will be remarked in this section.

All three transcripts were detected in seeds 24 hpi (Fig. 5a). In 3-, 6- and 10-d-old dark-grown seedlings *ST1*, *ST2* and *ST3* transcripts were detected both in aerial organs (namely hypocotyl and cotyledon) and in roots, except for *ST1* transcripts that were not found in the aerial part of 10-d-old etiolated seedlings (Fig. 5a). The amount of transcripts was, in general, higher in roots than in the aerial parts of the plant. The maximum transcript levels were detected in 3-d-old radicles and decreased with age although at different rates: either progressively (*ST1*), abruptly (*ST2*) or delayed (*ST3*), with the transcript levels in 6-d-old seedlings being as high as those in 3-d-old seedlings (Fig. 5a).

A higher level of *ST* transcript accumulation was observed in light-grown plants (Fig. 5b) as compared to the dark-grown plants. Maximum transcript levels were also found in the roots of 3-d-old seedlings, being more noticeable for *ST1* and *ST2*, while the levels of *ST3* also remained high in the other organs/stages analysed (Fig. 5b). Differences in transcript accumulation, when comparing root and aerial parts, were found among the *STs*. *ST1* accumulation was very similar between both parts except in 3-d-old seedlings; *ST2* was clearly higher in roots in the time lapse analysed and *ST3* displayed similar levels in roots and aerial parts (Fig. 5b).

Accumulation of transcripts in the roots, leaves, flowers and pods of mature plants, as well as in the late filling stage of seeds (green seed), were also investigated (Fig. 5c and amplification 5c1). Transcript levels at 3 different stages of leaf, flower and pod development were compared (1 to 3 from youngest to oldest; Additional file 3). All transcripts, except *ST3*, were much more abundant in roots than in leaves, where they were barely detected (Fig. 5c1). *ST3* accumulated at a high level in small developing leaves (stage 1) decreasing dramatically afterwards (Fig. 5c).

*ST1*, *ST2* and *ST3* transcripts were detected throughout all the reproductive stages analysed with a similar pattern (Fig. 5c1). Along flower development small variations, not statistically significant, were observed (Fig. 5c1). However, along pod development the three *ST* transcripts especially accumulated in pod stage 3 and in green seeds (Fig. 5c).

**Fig. 6:**
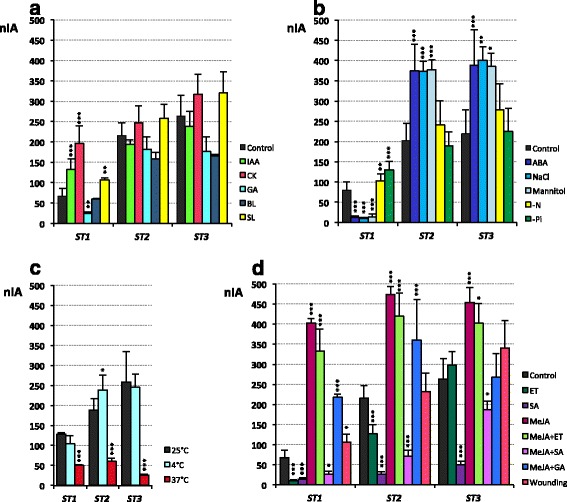
sqRT-PCR of *ST1*, *ST2* and *ST3* transcripts in *Medicago truncatula* seedlings under different treatments. **a** Transcript accumulation in untreated 7-d-old seedlings (control) and 7-d-old seedlings exposed during the last 24 h to indolacetic acid (IAA, 10 μM); benzylaminopurine (CK, 10 μM); giberellic acid (GA, 100 μM); epibrassinolide (BL, 10 μM) and strigolactone GR24 (SL, 10 μM). **b** Transcript accumulation in untreated 7-d-old seedlings (control) and 7-d-old seedlings exposed during the last 24 h to abscisic acid (ABA, 100 μM); sodium chloride (NaCl, 150 mM); mannitol (250 mM); N starvation (−N) plants where kept for 7 days in Fahräeus without N supplement and Pi starvation (−Pi) plants where kept for 7 days in Fahräeus-N without phosphate. **c** Transcript accumulation in plants exposed at different temperatures: 25 °C (control), −4 °C and 37 °C in darkness for the last 12 h in 7-d-old seedlings. **d**: Transcript accumulation in untreated 7-d-old seedlings (control) and 7-d-old seedlings exposed during the last 24 h to ethephon (ET, 1 mM); methyl jasmonate (MeJA, 100 μM); salicylic acid (SA, 1 mM); as well as a mixture of MeJA with one of the following: ET (MeJA + ET); SA (MeJA + SA) and GA (MeJA + GA) at the same concentrations as when individually applied. Wounding was performed in one foliole by transversally cutting the middle vein. Transcript accumulation was measured relative to ubiquitin as indicated in the Methods section. Scale units express normalized and integrated absorbance (nIA). Statistical analyses were conducted as stated in the Methods section considering 3 levels of significance: *p < 0.05; **p < 0.01; ***p < 0.001

In addition to the study of transcript levels throughout plant development, *ST* accumulation in 7-d-old seedlings exposed to different treatments was analysed (Fig. 6). We grouped the treatments taking into account the putative roles proposed for the ST proteins thus we considered those treatments mainly related to normal growth and development (Fig. 6a), ABA and abiotic stress situations (Fig. 6b) except different temperature conditions (Fig. 6c), and those whose participation in biotic interactions has been thoroughly established (Fig. 6d). Obviously, this is a broad classification owing to the complex interrelations between hormones and physiological processes (i.e. the role of strigolactones both in branching and in biotic interactions), but it helps to clarify and analyse the obtained results.

As *ST*s have been associated with elongation [3], we analysed the effect of hormones mainly related to normal growth and development in *ST* transcript accumulation (Fig. 6a). The results indicated that *ST1* behaved differently than *ST2* and *ST3*, which shared the same profile of mRNA levels. *ST1* transcripts were the only ones that significantly increased after IAA, CK and SL treatments, but were reduced by treatment with GA (Fig. 6a). *ST2* and *ST3* transcripts showed slight changes, such as a reduction in RNA levels by IAA, GA and BL and an increase in transcript accumulation by CK and SL, but none of these changes were statistically significant (Fig. 6a).

The second group of conditions tested was related to abiotic stress and included the study of: ABA; salt (NaCl) and osmotic (mannitol) stresses; nutrient starvation (N and Pi) (Fig. 6b); and exposure to unfavourable growth temperatures (4 °C and 37 °C) (Fig. 6c). When plants were grown in medium containing ABA, NaCl or mannitol, *ST1* transcript levels decreased dramatically, while *ST2* and *ST3* increased significantly (Fig. 6b). Nutritional starvation only clearly increased *ST1* accumulation (Fig. 6b). Finally, high temperatures had a significant inhibitory effect on *ST* transcript accumulation, while low temperatures had almost no effect (Fig. 6c).

To complete this study, the effect caused on *ST* transcript accumulation by hormones involved in responses to biotic stress (ET, MeJA and SA) and mechanical wounding (Fig. 6d) was analysed. These treatments produced statistically significant changes in the levels of all three *ST* transcripts, where *ST3* was the least affected. Generally, the application of ET and SA decreased *ST* transcript accumulation, except ET for *ST3*, while MeJA induced their levels (8 times for *ST1* and 2 times for *ST2* and *ST3*) (Fig. 6d). The effect of MeJA was slightly reduced when applied together with ET or with GA, and drastically reduced if a mixture of MeJA and SA was added to the growing trays (Fig. 6d). All of these results were in agreement with the individual effect of each hormone. Furthermore, mechanical wounding only significantly increased the transcript levels of *ST1,* although, not as intensely as the MeJA treatment.

### ST1 protein was located in the cell wall while ST2 and ST3 had a double location

**Fig. 7:**
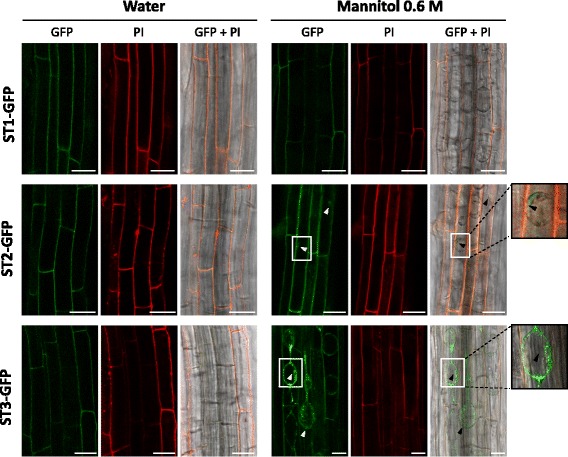
Subcellular location of *Medicago truncatula* ST1, ST2 and ST3 proteins in *Arabidopsis thaliana*. Confocal microscopy images of roots from Arabidopsis p35S::ST-GFP plants (ST1, ST2 and ST3) mounted in water or in 0.6 M mannitol. First column (GFP) show *green* fluorescence from ST-GFP fusion proteins, second column (PI) red fluorescence for propidium iodide stained cell walls and third column (GFP + PI) both channels merged with DIC channel. Bars = 25 μm

The cellular compartment where a protein is located determines the range of functions that it may perform. Thus, ST proteins fused to green fluorescent protein (GFP) at its C-terminal end were localized by confocal microscopy within root cells of transgenic *A. thaliana* (p35S::ST-GFP) (Fig. 7).

The results obtained when the roots were mounted in water (turgid cells) showed that the three ST proteins were localized at the cell boundaries and green fluorescence co-localized with red fluorescence from propidium iodide (PI) stained cell wall (Fig. 7). However, turgid cells made it difficult to distinguish whether the fusion ST-GFP proteins were clearly in the wall. To avoid this problem, roots were mounted in mannitol to cause cell plasmolysis and to separate the plasma membrane from the cell wall. In this case, ST1-GFP clearly co-localized with PI stained cell wall (Fig. 7). However, ST2 and ST3 were detected in the cell wall and also in the contracted cytoplasm, as deduced by the presence of green fluorescence inside the cell that did not co-localize with red fluorescence. This dual location was clearer in p35S::ST3-GFP plants (Fig. 7).

## Discussion

ST proteins were first described in pea [1, 2, 4] and subsequently characterized in chickpea [3]. The roles of these proteins, containing tandem repeats [2–5] and associated with the DUF2775 domain [23], remain elusive. *M. truncatula* has the largest *ST* gene family (6 *ST* genes) with a defined genome distribution, suggesting that some *MtST* genes may have arisen by duplication and then later became subfunctionalized or neofunctionalized (reviewed in [24]). The starting hypothesis of our studies regarding the function of *ST*s of *M. truncatula* was that each family member might have a specific role, and hence, that each *ST* gene might be subjected to a different type of regulation throughout plant development and/or in response to environmental factors. Accordingly, we have studied the regulation of the *M. truncatula ST* family by analysing their promoter regions (both in vivo and in silico), their transcript accumulation and the subcellular location of the proteins. The results obtained have enabled us to establish three functional groups among the whole family (data not shown). In this work we present two of them that include three members of the family, namely ST1, ST2 and ST3. ST1 protein is a type I ST, whose gene is located in chromosome 4, and ST2 and ST3 are type II STs, encoded by two consecutive genes in chromosome 3 [5].

The regulation of gene expression at the promoter level is mainly controlled by type, number, position and combination of CREs localized in the proximal zone, mainly in the first 500 bp upstream the TSS [25]. Furthermore, post-transcriptional regulation determines the accumulation of mRNA, and to some degree the amount of protein available within the cell. The activity pattern of several plant promoters is conserved even between angiosperm and gymnosperm [26], indicating the universal functionality of CREs, although differential performance has also been reported [27, 28]. Taking advantage of the conserved functionality of CREs among species, we have studied the *ST* gene promoters of *M. truncatula* using the model plant *Arabidopsis thaliana*.

Bioinformatics indicates that *pST1*, *pST2* and *pST3* have a TATA box determining the TSS. Although it is more common to find promoters without a TATA box in plant genes [29], its presence is not unusual in duplicated genes [30], as could be the case of *M. truncatula ST* family*.* The three *pSTs* analysed contain CREs that respond to endogenous factors (organ or tissue specific, hormones and enhancers) and environmental factors (light, abiotic stress and biotic interactions) (113 different motifs), although they differ in the combination and number of the different CREs (Fig. 1, Additional files 1 and 2). The amount of CREs found in each *pST* was very high (from 166 to 225 in the 1000 bp analysed) (Additional files 1 and 2) as often happens in in silico promoter analysis, given the small size of CREs (as an example a 4 nt CRE could appear once in 256 bp by chance). Consequently, only a small percentage of the CREs found in silico (around 5%) would constitute real binding sites for TFs and be functional [31]. Therefore, in this work, we will only discuss such CREs related to the in vivo promoter activity and the transcript accumulation analysed. The *ST* gene expression profiles coming from the microarray experiments and available at MtGEA were also considered [8, 9]. Although this information is of high value for the study of most transcripts, it should be carefully considered with respect to the highly conserved tandem repeat proteins, such as STs.

According to our results *ST1*, *ST2* and *ST3* profiles share several characteristics, mostly during vegetative development. Concerning *pST* activity, we are able to highlight several common features such as activity both in light and darkness, highest activity in youngest seedlings, predominant activity in roots, and activity associated with the vascular cylinder and leaf veins (Figs. 2, 3). The transcript accumulation pattern (Fig. 5) usually confirms the promoter activity profile (Figs. 2, 3) and allows us to contextualize our results. *ST1*, *ST2* and *ST3* transcripts are ubiquitously detected in almost all of the developmental stages analysed (Fig. 5), which could suggest that these ST proteins carry out their function throughout plant development.

*M. truncatula pST1*, *pST2* and *pST3* are active (Fig. 2) and transcripts accumulate (Fig. 5a, b) in seedlings growing in darkness and in light, in agreement with previous reports in pea and chickpea [1, 3]. However, while promoter activities are generally higher in etiolated seedlings, primarily in cotyledons (Fig. 2a), transcript levels show the contrary (Fig. 5a, b), which is opposite to *ST* transcript accumulation described in pea [2]. CREs associated with the control of gene expression by light are abundant in all *pST*s. On the one hand, the presence of elements driving transcription in darkness, ABRELATERD CRE (ABRE-like element required for expression of *erd1* in dark-induced senescence) mainly in *pST3* (6 times), SORLREP3AT (motif in light-repressed promoters) (1 in *pST3*), and REALPHALGLHCB21 (associated to an increase of expression in darkness) in *pST1* and *pST2* (Fig. 1b; Additional file 2), could explain the promoter activity and the mRNA accumulation in etiolated plants (Figs. 2, 5a). Additionally, typical LREs (as defined by Terzaghi and Cashmore [28]) are also present in *pST,* however no individual LRE can confer light-responsiveness on its own; it is their combination that ultimately determines the effect [28, 32]. The activity of all promoters in green adult plants could be explained by the presence of the typical LRE GT1CONSENSUS motif that confers light induction in mature plants, but not in seedlings [32].

Besides its usefulness in studying light responses, comparing green and etiolated seedlings is a good way to establish the relationship of a protein with elongation, one of the functions postulated for STs in chickpea [3, 7, 15, 20]. Here, the decrease of *pST* activity (Fig. 2a, c) and *ST* transcript accumulation (Fig. 5a, b) along plant growth, lead us to consider that barrel medic ST proteins are not related to elongation. Although certain data regarding *ST1* (the increase in *ST1* transcript accumulation by IAA, CK and SL [Fig. 6a]), could suggest the involvement of *ST1* in elongation, other data (the low transcript accumulation in etiolated seedlings with a high elongation rate [Fig. 5a], the decrease after GA application [Fig. 6a], or the strong induction of transcript accumulation by MeJA, a negative growth regulator [Fig. 6d] [33, 34]), cause us discard this function for ST1 as well.

Throughout plant development, higher *pST* activity and transcript accumulation in the earlier stages of vegetative organs (Fig. 2, 5), as well as in apical areas - mainly in *pST2* and *pST3* (Figs. 2, 3) -, is consistent with the presence of CREs associated with specific expression in meristematic zones such as NTBBF1ARROLB (Fig. 1b, Additional files 1 and 2). This CRE is also a vasculature-specific element (Additional file 2), which explains the other common features of *pST* activity, such as the strong association with vasculature in both aerial parts of the plant and in roots (Figs. 2, 3). Interestingly, CREs related to xylem are only found in *pST2* (Fig. 1b; Additional files 1 and 2).

*ST1*, *ST2* and *ST3* transcripts levels are, in general, higher in roots than in aerial parts (Fig. 5), similar to what occurs in chickpea radicles [3] and grapevine roots [16], but not in garden pea [2]. These results were also suggested from the EST profile [5] and MtGEA database [8, 9], and agree with the predicted percentages of root-related CREs (Fig. 1a, Additional file 1) and the activity of *pSTs* in roots (Figs. 2, 3). In fact, the high level of *pST2* activity in roots (Fig. 2b2, 3a) corresponds to the high number of root motifs present (9%), mainly ROOTMOTIFTAPOX1 (Fig. 1a; Additional files 1 and 2), allowing gene expression in the roots of adult plants (Additional file 2). In contrast to previous studies in *C. arietinum* [3, 20] and *P. sativum* [1, 2, 4], which showed a high transcript level of *STs* in etiolated epicotyls and stem internodes, no CREs related to stem, hypocotyl and/or epicotyl are found (Additional file 2), and no general promoter activity is detected in such etiolated organs (Fig. 2a).

Apart from the common features described above, our results showed fundamental differences among STs that can be divided into two main categories: reproductive development (Fig. 4) and responses to different treatments (Fig. 6). The putative function of STs during flower, fruit and seed development remains controversial depending on the species [3, 4, 16]. Barrel medic *pST* activity showed a highly specific pattern (Fig. 4), and few CREs associated with the reproductive stage have been found (Fig. 1a, b; Additional files 1 and 2). Based on these differences we decided to establish 2 functional groups: the first one includes ST1 and the second one includes ST2 and ST3.

*ST1* is the only gene studied whose promoter carries both co-dependent CREs related to late pollen-specific expression (POLLEN1LELAT52 and POLLEN2LELAT52), as well as GTGANTG10 (9 times) (Fig. 1b, Additional file 2), which correlates with in vivo *pST1* activity (Fig. 4) and *ST1* transcript accumulation in flower stages 1 and 2 (Fig. 5c1). During male gametophyte development there are genes that are expressed both early (undetectable in mature pollen) and late (accumulate as pollen matures) [35]. Therefore, the blue staining, driven by *pST1*, points to a late pollen-regulated expression (Fig. 4c1, d1), suggesting that the ST1 protein could accumulate in the male gametophyte, although, its putative role remains elusive.

The *M. truncatula* ST1 protein is very similar to *C. arietinum* ST1 (CarST1) as both are type I ST proteins, as defined by Albornos et al. [5]. Regardless of the number of ST proteins that a given species may have, there is always only one type I ST, which suggests the same conserved function for all of them*.* Since Albornos et al. [7] provide evidence describing CarST1 as a type of vegetative storage protein related to N storage and mobilization in both vegetative organs and in seeds, we also discuss this possibility for ST1 in *M. truncatula*. The fact that *ST1* transcript levels increase during N starvation (Fig. 6b), when N reserves should be metabolized, and the fact that etiolated cotyledons show *pST1* activity (Fig. 2), when storage proteins in cotyledons should be degraded to give N and carbon [36], are in disagreement with a role as reserve protein for barrel medic ST1. This is contrary to what has been described in chickpea, where no *CarST1* transcripts are found in etiolated cotyledons [3] and where protein levels decrease as germination proceeds [7]. These differences could be due to the different fates of the cotyledons of chickpea (hypogeal germination), Arabidopsis or barrel medic (epigeal germination), where in chickpea the cotyledons degenerate and in Arabidopsis or barrel medic the cotyledons behave as leaves. Also, it should be considered that ST1, unlike CarST1 [7], is clearly located only in the apoplast (Fig. 7) and not in protein bodies inside cells, the place where several reserve proteins accumulate [37].

However, although ST1 does not seem to be a reserve protein, we cannot discard that *pST1* may be regulated by the nutritional status of the plant, as *pST1* is the only one that contains the CRE AMMORESIIUDUNIA (Fig. 1c, Additional file 2), associated with genes involved in N metabolism, and also the CRE P1BS (Fig. 1c, Additional file 2), related to transcriptional activation of phosphate transporters induced in mycorrhizal symbiosis [38]. Different data support this hypothesis: *ST1* transcripts accumulation increases under N and Pi starvation (Fig. 6b); a high number of *ST1* ESTs were found in cDNA libraries made from plants establishing symbiosis with N fixing bacteria and arbuscular mycorrhizal fungi or under Pi starvation [5]; and three independent microarray experiments available at MtGEA (including [39]) indicate that *ST1* mRNA increases under N limiting conditions. Also supporting this idea, barrel medic *ST1* transcripts were strongly induced by MeJA (Fig. 6d), which has a major role in plant defence and alters N transport and distribution [40–42] since pathogen attack increases the sink force of an organ (reviewed in [43]). Finally, the *pST1* activity in funiculi (Fig. 4e1) supports its function in maternal and/or embryo nutrient transport and assimilation, as the funiculus provides a way for nutrients to move from the mother plant [44, 45].

In the second functional group, promoter activity and transcript accumulation point to a broad presence of ST2 and ST3 in the plant regardless of age, where they are more represented than ST1 in almost all conditions studied (Fig. 2-5). This result agrees with previous EST profile results that indicate that only *ST2* and *ST3* were detected in plants growing under normal conditions [5]. ST2 and ST3 show similar mRNA accumulation profiles, with high transcript accumulation in pods as well as in green seeds (Fig. 5c). This is in accordance with the fact that both *pST2* and *pST3* are active in seeds (Fig. 4d2, c3-g3), and with the presence of several seed-specific CREs within their sequences (Fig. 1a, b; Additional files 1 and 2), some of them found in promoters of genes encoding seed storage proteins (SSP). However, it has been checked that none of the combinations of CRE that usually drive the expression of SSP, such as B-box and RY-G-box in dicotyledonous (reviewed in [46]), are present either in *pST2* or in *pST3*. Moreover, there are other characteristics that allow us to discard that ST2 and ST3 are SSP, i.e., *pST2* and *pST3* promoters are active in developmental phases other than seed filling (Figs. 2, 3, 4) and transcripts accumulate all through plant development (Fig. 5).

Once the role of ST2 and ST3 as SSP has been discarded, the high level of *ST2* and *ST3* transcripts found in green seeds in the late filling phase, when storage accumulation still takes place and the water content diminishes by half and the desiccation tolerance increases exponentially [47], might relate these proteins with a physiological water deficit. Moreover, *ST2* and *ST3* transcripts are strongly induced by ABA, NaCl and mannitol (Fig. 6b), what lead us to consider a role in situations of physiological and/or environmental water deficit, resembling late embryogenesis abundant (LEA) proteins (reviewed by [48]). ST2 and ST3 amino acid sequence share characteristics with LEA proteins such as high hydrophobicity, high content of Gly and small amino acids like Ser or Ala, a high proportion of charged amino acids and the lack of Cys and Trp (reviewed by [48]). Furthermore, several LEA proteins have tandem repeats and could be intrinsically unstructured proteins [49–51], as has been pointed out for ST2 and ST3 [5]. The promoter activity pattern (Figs. 2, 3) and the transcript accumulation profile (Figs. 5, 6) indicate that ST2 and ST3 proteins could show a similar distribution as some of the LEA proteins, not only under dehydration conditions but also during plant normal growth [52–54]. Thus, the accumulation of *ST3* transcripts in leaves at early developmental stages (Fig. 5c1) as occurs for several LEA proteins [53], the *pST2* activation in apical root (Fig. 2a2, b3, 3a2) where numerous LEA proteins accumulate [52], the presence of both STs and LEA in mature organs such as roots and flower [52], and finally, the accumulation of LEA proteins in pollen grains [55] where *pST3* transiently activates (Fig. 4a3), support this notion. Also, *pST3* has a CRE motif associated with ABA-regulated late embryogenesis expression (DRE2COREZMRAB17; Additional file 2).

All of these similarities between ST2 and ST3 and LEA proteins suggest that ST2 and ST3 could play a role in response to water deficit. Intracellular accumulation of ST2 and ST3 is compatible with this function. In fact, the subcellular location of ST2 and, mainly ST3, can contribute to membrane stability when the cell suffers an osmotic shock (Fig. 7). This role in dehydration is supported by the presence of specific CREs in their promoters. TFs that modulate gene expression in situations of abiotic stress bind to ABA responsive elements (ABRE) in ABA-dependent responses. In this sense, *pST2* and *pST3* contain 3 and 4 ABRE sequences, respectively (ABRERATCAL, Fig. 1c; Additional files 1 and 2). They also contain G-boxes (CACGTGMOTIF; Additional file 2), which are coupling elements necessary for induction of gene expression in response to ABA and other stimulus [56–58], and MYB and MYC TF binding sites (13 and 15 motifs, respectively), that coordinate regulation of gene expression in abiotic stress (reviewed in [59, 60]). W-box elements, binding site of WRKY TF, were also found in *pST2* and *pST3* (Additional file 2). Although traditionally associated to biotic stress, WRKY TF have also a relevant role in coordinating responses to abiotic stress [61–63]. The induction of WRKY TF mediated by MeJA in response to water deficit has been determined [64], where the increase of *ST2* and *ST3* accumulation in plants treated with this hormone (Fig. 6d) is noteworthy.

A previous analysis of the EST profile [5] also supports the role of ST2 and ST3 in response to dehydration and other abiotic stresses, since their transcripts accumulate in situations of drought and salinity, as was found in the microarray assays collected in MtGEA [39]. Also, transcriptomic analysis in *Trifolium repens* shows an increase in *ST* transcripts when drought-tolerant plants grow under water stress [65]. By contrast, water stress decreases *ST* transcript accumulation in chickpea seedlings [3, 15] as occurs in *M. truncatula ST1* (Fig. 6b). This confirms that the various STs behave differently under abiotic stress.

## Conclusions

The ST proteins studied in this work, ST1, ST2 and ST3, are ubiquitous proteins that could perform different/complementary roles in plant biology as they are encoded by genes differentially regulated. The analysed *pSTs* show different combinations of specific CREs, and they also present different activity patterns. However, all *pSTs* were rich in tissue/organ specific CREs with the highest number representing those specific to roots and seeds. Common features in promoter activity and also in transcript accumulation have been found, mainly during vegetative development, such as the abundance in roots and in the vascular cylinder. Nevertheless, significant differences, mainly during reproductive development and in response to different stimuli, led us to consider two functional groups, which confirms our initial hypothesis. ST1 may participate in processes affected by nutritional status, while ST2 and ST3 seem to act when plants are challenged with abiotic stresses related to water stress and in physiologically controlled desiccation processes such as the seed maturation.

## Methods

### Plant material and growth conditions

*Arabidopsis thaliana* ecotype Columbia-0 (Col-0) was used as the host for transformations. Seeds were sterilized, stratified and germinated in Petri dishes on one-half-strength Murashige and Skoog (MS) [66] agar medium and 1% (*w*/*v*) sucrose, as described in Albornos et al. [67].

These seedlings were maintained in Petri dishes up to 10 days and then transferred to plastic pots containing a 3:1 mixture of potting soil and vermiculite. They were grown in a growth chamber (Aralab, Portugal) at 22 °C with a 16 h/8 h light/dark photoperiod (light provided by cool-white fluorescent tubes at a light intensity of approximately 80–100 μE/m^2^/s). For the analysis of promoter activity, seeds were either maintained in darkness at 25 °C (dark-grown plants), or in chambers as indicated above (light-grown plants), and collected after 3 and 10 d. Light-grown plants were transferred to pots at 12 d, and different organs were collected throughout development in order to perform GUS staining.

*Medicago truncatula* ecotype Jemalong line A17 seeds were chemically scarified with H_2_SO_4_ 95% (*v*/v), surface sterilized with 5% chlorine solution for 2 min, placed in Petri dishes with modified Fahräeus supplemented with NH_4_NO_3_ to a final 1 mM N concentration (Fahräeus-N) [68], and stratified for 2 d at 4 °C in the dark before germination.

For growing plants in pots, seeds were transferred to a mixture of soil:vermiculite (3:1), and grown in a chamber (Aralab, Portugal) at 25 °C with a 16 h/8 h light/dark photoperiod (light provided by a mix of cool-white and red fluorescent tubes at a light intensity of approximately 200–300 μE/m^2^/s).

For growing plants in trays, seeds were germinated on a glass plate covered with filter paper soaked in Fahräeus-N liquid media, either in the dark (dark-grown plants) or in a chamber using the above mentioned light conditions (light-grown plants).

To perform sqRT-PCR experiments, plants were grown in the light and in darkness in trays as indicated above, and collected at 24 hpi and at 3-, 6- and 10-d-old. Roots and aerial parts were collected separately, except for 24 hpi seeds, and immediately frozen in liquid N. Different organs (roots from 30-d-old plants; leaves, flowers and pods at 3 developmental stages; and green seeds) were collected from plants grown in pots upon maturity and immediately frozen in liquid N. The developmental stages of flowers and pods are based on Kurdyukov et al. [69], and all of the stages analysed are fully described in Additional file 3. Green seeds were collected at the end of the filling phase (24–26 d after pollination) according to Verdier et al. [47]. At that time, the water content in seeds has decreased below 50% and desiccation tolerance is increasing exponentially [47].

For the chemical treatments, germinated seeds were kept in trays with Fahräeus-N for 6 d at the usual conditions of light and temperature, and then transferred to a new tray for 24 h with Fahräeus-N supplemented with: abscisic acid (ABA, 100 μM); benzylaminopurine (CK, 10 μM); epibrassinolide (BL, 10 μM); ethephon (ET, 1 mM); giberellic acid (GA, 100 μM); indolacetic acid (IAA, 10 μM); mannitol (250 mM); methyl jasmonate (MeJA, 100 μM); salicylic acid (SA, 1 mM); sodium chloride (NaCl, 150 mM); strigolactone GR24 (SL, 10 μM); as well as a mixture of MeJA with one of the following: ET, GA and SA, at the same concentrations as when individually applied. Temperature treatment was carried out for 12 h in darkness at 4, 25 and 37 °C, with 25 °C as the control temperature. Finally, for N (−N) and Pi (−Pi) starvations, plants where kept for 7 days either in Fahräeus without N supplement or Fahräeus-N media without phosphate, respectively. Wounding was performed on one foliole by transversally cutting the middle vein.

### Cis-regulatory element search and promoter analysis

The promoters of three *M. truncatula ST* genes (*pST1*, *pST2* and *pST3*) were examined. Analyses were conducted using 1000 bp upstream of the ATG start codon of each gene. The transcript initiation site and the presence or absence of TATA boxes within the *pST* promoters were studied with the TSSP Software (Softberry Inc) using the default parameters. Searches for known CREs were conducted using PLACE [70] and PlantCARE [71]. Elements found in both direct and complementary chains of the DNA sequences were considered. All elements retrieved in the searches were taken into account except for the TATA boxes, matrix/scaffold attachment regions or TATA-less promoters transcription initiation motifs. Databases were scanned in the order shown above, and only non-redundant elements were considered for PlantCARE. Core elements were only considered when not overlapping with another specific CRE. The percentage of each category was calculated with respect to the total number of CREs found per promoter.

### Promoter and ORF cloning

A search for the *M. truncatula ST* gene family promoter region sequences was done using phytozome v9.1. The promoter region of each *ST* gene analysed, consisting of a sequence of ca. 2100–3000 bp upstream from the translational start site, was PCR-amplified (*Kapa HiFi HotStart polymerase* by Kapa Biosystems, USA) from *M. truncatula* gDNA, adding the *att*B1 and *att*B2 sequences to the 5′- and 3′-terminals for Gateway™ cloning. Additionally, a shorter version of *pST3* (*pST3*·F2 of 1073 bp) was cloned because in this version of the *M. truncatula* genome sequence there was a putative ORF 1 kb upstream of the *ST3* ORF. To obtain gDNA, the leaves of 40-d-old plants grown in pots were collected, immediately frozen in liquid N and ground using a MM 400 Mixer Mill (RETSCH, Germany). Two hundred mg of the ground sample were used to purify gDNA using Qiagen DNeasy Plant Mini kit (Qiagen, USA).

To clone *ST1* [EMBL:LN827607], *ST2* [EMBL:LN827608] and *ST3* [EMBL:LN827609] ORFs, RNA was extracted from roots. One hundred mg of each sample, obtained as indicated above, were used to obtain RNA using Nucleospin® RNA plant kit (Macherey-Nagel, Germany). To avoid RNA degradation all instruments were deep washed with the RNase inhibitor RNaseZap® solution (Ambion, USA).

Several primer pairs were designed for each promoter and each ORF using the online tool at [72] and those that allowed the cloning of the promoters (primers 1 to 7) and the ORF (primers 8 to 14) are listed in Additional file 5. The amplified products were gel-purified (NucleoSpin® Gel and PCR Clean-up by Macherey-Nagel, Germany), checked for the correct size and sequenced. Several attempts were made to clone the *ST1* ORF because the size of the expected PCR product, based on the deposited sequence, and the size of the obtained fragment were different. In fact an extra primer from 3′ UTR was used (number 3 in Additional file 5) in order to assure that the right *ST1* ORF had been cloned.

### *Arabidopsis thaliana* Transformation

The *pST::GUS* and *p35S::ST-GFP* reporter gene cassettes were prepared using Gateway™ cloning technology (Invitrogen, USA) according to the manufacturer’s instructions.

The cloned regions of each *ST* were used in the BP reaction with pDONR201 (Invitrogen, USA). pENTR201.*pST* and pENTR201.*ST* vectors were selected by PCR (primers 15 and 16 in Additional file 5), and sequenced to check for both correct cloning and sequence. The entry clones generated with cloned *pST* or ORF were used in the LR reaction with pKGWFS7, a C-terminal GFP/GUS destination vector, and pK7FWG2, a vector that generated GFP-fused proteins at C-terminal end driven by *p35S*, respectively, to make the corresponding expression vectors (for more characteristics see [73]) [74]. All transgene cassettes in expression vectors were verified by PCR using two pairs of primers, one of them that annealed in the cloned sequence (primers 1 to 14, Additional file 5) and the other in the vector (primers 17 and 18, Additional file 5).

All expression vectors generated were electroporated into *Agrobacterium tumefaciens* strain C58C1m, and the *A. thaliana* ecotype Col-0 was transformed using the *Agrobacterium*-mediated floral dip method [75].

Seeds harvested from infiltrated plants were screened using the appropriate antibiotic and resistant seedlings (T1) were selected. T2 plants were used for analysing promoter activities by the GUS assay and subcellular ST localization by confocal microscopy.

### GUS staining

GUS staining using X-glucuronide (5-bromo-4-chloro-3-indolyl-b-D-GlcUA) (Duchefa, The Netherlands) was performed as described by Albornos et al. [67].

Plant material was incubated overnight at 37 °C in GUS staining solution (5-bromo-4-chloro-3-indolyl-β-D-GlcA, 50 mM Na phosphate buffer pH 7.0, 2 mM potassium ferrocyanide, 2 mM potassium ferricyanide and 0.2% Triton X-100) and GUS-stained tissues were cleared in 70% ethanol.

GUS activity was assayed in 3 and 10-d-old seedlings grown on MS plates (both light- and dark-grown seedlings) and in the different organs of 12 to 38-d-old plants growing in the potting soil and vermiculite mixture, as already mentioned. Images were acquired using a Leica M205 FA stereomicroscope equipped with a Leica DFC495 camera (Leica Microsystems, Germany).

Experiments were performed in at least two plants of three independent T2 lines. Only when several independent transgenic lines displayed the same pattern of expression of the reporter gene was it consider as positive. Wild-type control plants showed no GUS activity (data not shown).

### Semi quantitative reverse-transcription polymerase chain reaction (sqRT-PCR)

First-strand complementary DNA (cDNA) was synthesized from 500 ng of RNA extracted as indicated above by priming with a mixture of hexanucleotides (final concentration 20 μM) plus oligo dT (final concentration 2.5 μM) using PrimerScript™ RT reagent kit (Takara Bio Inc.) following the manufacturer’s instructions. Once obtained, the cDNA was diluted 10 fold before using in the PCR analysis.

Because of the high sequence similarity existing among the *ST* genes, it was necessary to verify that the primers designed, flanking the intron sequence found in *ST* genes, only recognized the transcript of interest. Several primer pairs were tested in both gDNA and cDNA, as well as in the ORF previously cloned. The specific primers used are listed in Additional file 5 (numbers 19 to 24). A phosphatidylinositol 3- and 4-kinase belonging to ubiquitin family (Medtr3g091400 gene, TC102473) tested by Kakar et al. [76] was used as an internal standard, and the PCR primers used for its amplification are also listed in Additional file 5 (numbers 25 and 26). The fragments amplified using cDNA as a template ranged from 150 to 250 pb.

PCR was performed by GoTaq® DNA polymerase (Promega, USA) using 2 μl of 1:10 diluted cDNA in a standardized protocol of: 1 × 95 °C for 5 min; 32 × 95 °C for 1 min, 60 °C for 1 min, 72 °C for 30 s; and 1 × 72 °C for 5 min. Images of the ethidium bromide-stained agarose gels were acquired with a ChemLite 400 FA (Avegene Life Science, Taiwan) (Additional file 4) and quantification of the bands was performed by TotalLab Quant 1D gel analysis v.10. Quantification of ST amplicons was done relative to the ubiquitin band, which was given an arbitrary value of 100 units. Band intensity was expressed as normalized and integrated absorbance (nIA). RNA was obtained from two independent biological replicates, and two cDNA synthesis reactions of each RNA were performed. Every cDNA template was used for sqPCR at least twice. Mean and standard deviation of all experiments were calculated. Statistical analyses were carried out using IBM SPSS statistics v23. When comparing transcript levels of a given *ST* gene among organs and developmental stages a one-way ANOVA followed by Scheffe’s test was performed, significant variations were considered for *p*-values less than 0.05. When treatments were compared to a control, ANOVA followed by Dunnett’s test were performed to find variations. Significant variations were considered for *p*-values less than 0.05, being: * 95% confidence level (*p*-value < 0.05); ** 99% confidence level (*p*-value < 0.001); and *** 99.9% confidence level (*p*-value < 0.05).

### Confocal microscopy

Roots of 22 to 25-d-old T2 transgenic plants having *ST1*, *ST2* or *ST3* ORFs fused to GFP were observed by confocal microscopy (LEICA DMI-6000B equipped with confocal SP5 system). Before visualization roots were mounted either in water or in 0.6 M mannitol in order to plasmolyse the cells. Cell wall staining was performed by immersing the roots in a 10 μg/ml PI solution for 2 min, followed by extensive rinsing with distilled water.

## Additional files

Additional file 1: Classification of *cis*-acting regulatory elements found in *ST1, ST2* and *ST3* promoters under different categories and subcategories. (DOCX 29 kb)

Additional file 2: List of all *cis*-acting regulatory elements found in *pST1*, *pST2* and *pST3* promoters using the PLACE and the PlantCARE databases. (XLSX, 29 kb)

Additional file 3: *Medicago truncatula* plants along its development. (PDF 3399 kb)

Additional file 4: Agarose (2%) gel electrophoresis of sqRT-PCR products. (PDF 850 kb)

Additional file 5: Primers used in PCR amplifications. (DOCX 78 kb)

## Abbreviations

ABRE: ABA responsive element
Arabidopsis: *Arabidopsis thaliana*
CRE: *Cis*-acting regulatory element
DUF: Domain of unknown function
EST: Expressed sequence tag
gDNA: Genomic DNA
GFP: Green fluorescent protein
GUS: β-glucuronidase
hpi: Hours post imbibition
LEA: Late embryogenesis abundant
LRE: Light regulated elements
MS: Murashige and Skoog media
MtGEA: *Medicago truncatula* gene expression atlas
NIA: Normalized and integrated absorbance
ORF: Open reading frame
p35S::ST-GFP: Transgenic Arabidopsis plants carrying *p35S::ST-GFP* transgene
PI: Propidium iodide
*pST*: Promoter of *Medicago truncatula ST* gene
pST::GUS: Transgenic Arabidopsis plants carrying *pST::GUS* transgene
sqRT-PCR: Semi quantitative reverse-transcription polymerase chain reaction
SSP: Seed storage protein
ST: ShooT specific/Specific Tissue
TF: Transcription factor
TSS: Transcription start site

## References

[1] De Vries SC, Harmsen MC, Kuiper MTR, Dons HJM, Wessels JGH (1983). Molecular cloning of pea mRNAs encoding a shoot-specific polypeptide and light-induced polypeptides. Plant Mol Biol.

[2] De Vries SC, De Vos WM, Harmsen MC, Wessels JGH (1985). A shoot-specific mRNA from pea: nucleotide sequence and regulation as compared to light-induced mRNAs. Plant Mol Biol.

[3] Muñoz FJ, Dopico B, Labrador E (1997). Two growth-related organ-specific cDNAs from *Cicer arietinum* epicotyls. Plant Mol Biol.

[4] Williams ME, Mundy J, Kay SA, Chua N-H (1990). Differential expression of two related organ-specific genes in pea. Plant Mol Biol.

[5] Albornos L, Martín I, Iglesias R, Jiménez T, Labrador E, Dopico B (2012). ST proteins, a new family of plant tandem repeat proteins with a DUF2775 domain mainly found in Fabaceae and Asteraceae. BMC Plant Biol.

[6] Katti MV, Sami-Subbu R, Ranjekar PK, Gupta VS (2000). Amino acid repeat patterns in protein sequences: their diversity and structural-functional implications. Prot Sci.

[7] Albornos L, Cabrera J, Hernández-Nistal J, Martín I, Labrador E, Dopico B (2014). Organ accumulation and subcellular location of *Cicer arietinum* ST1 protein. Plant Sci.

[8] Benedito V, Torres-Jerez I, Murray J, Andriankaja A, Allen S, Kakar K, Wandrey M, Verdier J, Zuber H, Ott T, Moreau S, Niebel A, Frickey TD, Weiller G, He J, Dai X, Zhao P, Tang Y, Udvardi M (2008). A gene expression atlas of the model legume *Medicago truncatula*. Plant J.

[9] He J, Benedito VA, Wang M, Murray JD, Zhao PX, Tang Y, Udvardi MK (2009). The *Medicago truncatula* gene expression atlas web server. BMC Bioinformatics.

[10] Gaude N, Bortfeld S, Duensing N, Lohse M, Krajinski F (2012). Arbuscule-containing and non-colonized cortical cells of mycorrhizal roots undergo a massive and specific reprogramming during arbuscular mycorrhizal development. Plant J.

[11] Kuhn H, Küster H, Requena N (2009). Membrane steroid-binding protein 1 induced by a diffusible fungal signal is critical for mycorrhization in *Medicago truncatula*. New Phytol.

[12] Liu J, Maldonado-Mendoza I, López-Meyer M, Cheung F, Town CD, Harrison MJ (2007). Arbuscular mycorrhizal simbiosis in gene expression and an increase in disease resistance in the shoots. Plant J.

[13] Maillet F, Poinsot V, André O, Puech-Pagès V, Haouy A, Gueunier M, Cromer L, Giraudet D, Formey D, Niebel A, Martinez EA, Driguez H, Bécard G, Dénarié J (2011). Fungal lipochitooligosaccharide symbiotic signals in arbuscular mycorrhiza. Nature.

[14] Wulf A, Manthey K, Doll J, Perlick AM, Linke B, Bekel T, Meyer F. Franken P, Küster H, Krajinski F. Transcriptional changes in response to arbuscular mycorrhiza development in the model plant *Medicago truncatula.* Mol Plant-Microbe Interact 2003;16:306-314. 10.1094/MPMI.2003.16.4.306 12744459

[15] Hernández-Nistal J, Martín I, Esteban R, Dopico B, Labrador E (2010). Abscisic acid delays chickpea germination by inhibiting water uptake and down-regulating genes encoding cell wall remodelling proteins. Plant Growth Reg.

[16] Fernandez L, Torregrosa L, Terrier N, Sreekantan L, Grimplet J, Davies C, Thomas MR, Romieu C, Ageorge A (2007). Identification of genes associated with flesh morphogenesis during grapevine fruit development. Plant Mol Biol.

[17] Levi A, Davis A, Hernandez A, Wechter P, Thimmapuram J, Trebitsh T, Tadmor Y, Katzir N, Portnoy V, King S (2006). Genes expressed during the development and ripening of watermelon fruit. Plant Cell Rep.

[18] Waters DLE, Holton TA, Ablett EM, Lee LS, Henry RJ (2005). cDNA microarray analysis of developing grape (*Vitis vinifera* cv. Shiraz) berry skin. Funct Integr Gen.

[19] Wechter WP, Levi A, Harris KR, Davis AR, Fei Z, Katzir N, Giovannoni JJ, Salman-Minkov A, Hernandez A, Thimmapuram J, Tadmor Y, Portnoy V, Trebitsh T (2008). Gene expression in developing watermelon fruit. BMC Gen..

[20] Hernández-Nistal J, Labrador E, Martín I, Jiménez T, Dopico B (2006). Transcriptional profiling of cell wall protein genes in chickpea embryonic axes during germination and growth. Plant Physiol Biochem.

[21] Varshney RK, Song C, Saxena RK, Azam S, Yu S, Sharpe AG, Cannon S, Baek J, Rosen BD, Tar'an B, Millan T, Zhang X, Ramsay LD, Iwata A, Wang Y, Nelson W, Farmer AD, Gaur PM, Soderlund C, Penmetsa RV, Xu C, Bharti AK, He W, Winter P, Zhao S, Hane JK, Carrasquilla-Garcia N, Condie JA, Upadhyaya HD, Luo MC (2013). Draft genome sequence of chickpea (*Cicer arietinum*) provides a resource for trait improvement. Nat Biotech.

[22] Tang H, Krishnakumar V, Bidwell S, Rosen B, Chan A, Zhou S, Gentzbittel L, Childs KL, Yandell M, Gundlach H, Mayer KF, Schwartz DC, Town CD (2014). An improved genome release (version Mt4.0) for the model legume *Medicago truncatula*. BMC Gen.

[23] Marchler-Bauer A, Lu S, Anderson JB, Chitsaz F, Derbyshire MK, DeWeese-Scott C, Fong JH, Geer LY, Geer RC, Gonzales NR, Gwadz M, Hurwitz DI, Jackson JD, Ke Z, Lanczycki CJ, Lu F, Marchler GH, Mullokandov M, Omelchenko MV, Robertson CL, Song JS, Thanki N, Yamashita RA, Zhang D, Zhang N, Zheng C, Bryant SH (2011). CDD: a conserved domain database for the functional annotation of proteins. Nucleic Acids Res.

[24] Koonin EV (2005). Orthologs, paralogs, and evolutionary genomics. Annu Rev Genet.

[25] Fauteux F, Strömvik MV (2009). Seed storage protein gene promoters contain conserved DNA motifs in *Brassicaceae*, *Fabaceae* and *Poaceae*. BMC Plant Biol.

[26] Germain H, Lachance D, Pelletier G, Fossdal CG, Solheim H, Séguin A (2012). The expression pattern of the *Picea glauca Defensin 1* promoter is maintained in *Arabidopsis thaliana*, indicating the conservation of signalling pathways between angiosperms and gymnosperms. J Exp Bot.

[27] Hernandez-Garcia CM, Finer JJ (2014). Identification and validation of promoters and *cis*-acting regulatory elements. Plant Sci.

[28] Terzaghi WB, Cashmore AR (1995). Light-regulated transcription. Annu Rev Plant Physiol Plant Mol Biol.

[29] Morton T, Petricka J, Corcoran DL, Li S, Winter CM, Carda A, Benfey PN, Ohler U, Megraw M (2014). Paired-end analysis of transcription start sites in *Arabidopsis* reveals plant-specific promoter signatures. Plant Cell.

[30] Zou Y, Huang W, Gu Z, Gu X (2011). Predominant gain of promoter TATA box after gene duplication associated with stress responses. Mol Biol Evol.

[31] Hardison RC, Taylor J (2012). Genomic approaches towards finding *cis*-regulatory modules in animals. Nature Rev Gen.

[32] Argüello-Astorga GR, Herrera-Estrella LR (1996). Ancestral multipartite units in light-responsive plant promoters have structural features correlating with specific phototransduction pathways. Plant Physiol.

[33] Yamane H, Sugawara J, Suzuki Y, Shimamura E, Takahashi N (1980). Syntheses of jasmonic acid related compounds and their structure-activity relationships on the growth of rice seedlings. Agric Biol Chem.

[34] Ueda J, Kato J (1982). Inhibition of cytokinin-induced plant growth by jasmonic acid and its methyl ester. Physiol Plant.

[35] McCormick S (1993). Male gametophyte development. Plant Cell.

[36] Patton DA, Meinke DW (1990). Ultrastructure of arrested embryos from lethal mutants of *Arabidopsis thaliana*. Am J Bot.

[37] Staswick PE (1994). Storage proteins of vegetative plant tissues. Annu Rev Plant Biol.

[38] Chen A, Gu M, Sun S, Zhu L, Hong S, Xu G (2011). Identification of two conserved cis-acting elements, MYCS and P1BS, involved in the regulation of mycorrhiza-activated phosphate transporters in eudicot species. New Phytol.

[39] Ruffel S, Freixes S, Balzergue S, Tillard P, Jeudy C, Martin-Magniette ML, van der Merwe MJ, Kakar K, Gouzy J, Fernie AR, Udvardi M, Salon C, Gojon A, Lepetit M (2008). Systemic signaling of the plant nitrogen status triggers specific transcriptome responses depending on the nitrogen source in *Medicago truncatula*. Plant Physiol.

[40] Beardmore T, Wetzel S, Kalous M (2000). Interactions of airborne methyl jasmonate with vegetative storage protein gene and protein accumulation and biomass partitioning in populus plants. Can J For Res.

[41] Rossato L, Le Dantec C, Laine P, Ourry A (2002). Nitrogen storage and remobilization in *Brassica napus* L. during the growth cycle: identification, characterization and immunolocalization of a putative taproot storage glycoprotein. J Exp Bot.

[42] Meuriot F, Noquet C, Avice J, Volenec JJ, Cunningham SM, Sors TG, Caillot S, Ourry A (2004). Methyl jasmonate alters N partitioning, N reserves accumulation and induces gene expression of a 32-kDa vegetative storage protein that possesses chitinase activity in *Medicago sativa* taproots. Physiol Plant.

[43] Schultz JC, Appel HM, Ferrieri AP, Arnold TM (2013). Flexible resource allocation during plant defense responses. Front Plant Sci.

[44] Meinke DW. Seed development in *Arabidopsis thaliana*. Cold Spring Harbor Monograph Series. 1994;27:253. http://cshmonographs.org/index.php/monographs/article/view/3103/2457.

[45] Schultz P, Jensen WA (1971). *Capsella* embryogenesis: the chalazal proliferating tissue. J Cell Sci.

[46] Vicente-Carbajosa J, Carbonero P (2005). Seed maturation: developing an intrusive phase to accomplish a quiescent state. Int J Dev Biol.

[47] Verdier J, Lalanne D, Pelletier S, Torres-Jerez I, Righetti K, Bandyopadhyay K, Leprince O, Chatelain E, Ly Vu B, Gouzy J, Gamas P, Udvardi MK, Buitink J (2013). A regulatory network-based approach dissects late maturation processes related to the acquisition of desiccation tolerance and longevity of *Medicago truncatula* seeds. Plant Physiol.

[48] Battaglia M, Olvera-Carrillo Y, Garciarrubio A, Campos F, Covarrubias AA (2008). The enigmatic LEA proteins and other hydrophilins. Plant Physiol.

[49] Lisse T, Bartels D, Kalbitzer HR, Jaenicke R (1996). The recombinant dehydrin-like desiccation stress protein from the resurrection plant *Craterostigma plantagineum* displays no defined three-dimensional structure in its native state. Biol Chem.

[50] Thalhammer A, Hundertmark M, Popova AV, Seckler R, Hincha DK (2010). Interaction of two intrinsically disordered plant stress proteins (COR15A and COR15B) with lipid membranes in the dry state. Biochim Biophys Acta-Biomembranes.

[51] Boucher V, Buitink J, Lin X, Boudet J, Hoekstra FA, Hundertmark M, Renard D, Leprince O (2010). MtPM25 is an atypical hydrophobic late embryogenesis-abundant protein that dissociates cold and desiccation-aggregated proteins. Plant Cell Environ.

[52] Nylander M, Svensson J, Palva ET, Welin BV (2001). Stress-induced accumulation and tissue-specific localization of dehydrins in *Arabidopsis thaliana*. Plant Mol Biol.

[53] Rorat T, Grygorowicz W, Irzykowski W, Rey P (2004). Expression of KS-type dehydrins is primarily regulated by factors related to organ type and leaf developmental stage during vegetative growth. Planta.

[54] Hundertmark M, Hincha DK (2008). LEA (late embryogenesis abundant) proteins and their encoding genes in *Arabidopsis thaliana*. BMC Gen..

[55] Wolkers WF, McCready S, Brandt WF, Lindsey GG, Hoekstra FA (2001). Isolation and characterization of a D-7 LEA protein from pollen that stabilizes glasses in vitro. Biochim Biophys Acta-Prot Struct Mol Enzymol.

[56] Donald RG, Cashmore AR (1990). Mutation of either G box or I box sequences profoundly affects expression from the arabidopsis rbcS-1A promoter. EMBO J.

[57] Kim SR, Choi JL, Costa MA, An G (1992). Identification of G-box sequence as an essential element for methyl jasmonate response of potato proteinase inhibitor II promoter. Plant Physiol.

[58] Shen Q, Ho TH (1995). Functional dissection of an abscisic acid (ABA)-inducible gene reveals two independent ABA-responsive complexes each containing a G-box and a novel cis-acting element. Plant Cell.

[59] Shinozaki K, Yamaguchi-Shinozaki K, Seki M (2003). Regulatory network of gene expression in the drought and cold stress responses. Curr Opin Plant Biol.

[60] Yamaguchi-Shinozaki K, Shinozaki K (2006). Transcriptional regulatory networks in cellular responses and tolerance to dehydration and cold stresses. Annu Rev Plant Biol.

[61] Zhou Q, Tian A, Zou H, Xie Z, Lei G, Huang J, Wang C, Wang H, Zhang J, Chen S (2008). Soybean WRKY-type transcription factor genes, GmWRKY13, GmWRKY21, and GmWRKY54, confer differential tolerance to abiotic stresses in transgenic arabidopsis plants. Plant Biotech J.

[62] Qiu Y, Yu D (2009). Over-expression of the stress-induced OsWRKY45 enhances disease resistance and drought tolerance in arabidopsis. Environ Exp Bot.

[63] Tripathi P, Rabara RC, Rushton PJ (2014). A systems biology perspective on the role of WRKY transcription factors in drought responses in plants. Planta.

[64] Rabara RC, Tripathi P, Lin J, Rushton PJ (2013). Dehydration-induced WRKY genes from tobacco and soybean respond to jasmonic acid treatments in BY-2 cell culture. Biochem Biophys Res Commun.

[65] Yates SA, Swain MT, Hegarty MJ, Chernukin I, Lowe M, Allison GG, Ruttink T, Abberton MT, Jenkins G, Skot L (2014). De novo assembly of red clover transcriptome based on RNA-Seq data provides insight into drought response, gene discovery and marker identification. BMC Gen.

[66] Murashige T, Skoog F (1962). A revised medium for rapid growth and bioassays with tobacco tissue cultures. Physiol Plant.

[67] Albornos L, Martín I, Pérez P, Marcos R, Dopico B, Labrador E (2012). Promoter activities of genes encoding β-galactosidases from Arabidopsis a1 subfamily. Plant Physiol Biochem.

[68] Barker DG, Faff T, Moreau D, Groves E, Ruffel S, Lepetit M, Whitehand S, Maillet F, Nair, RM, Journet E. Growing *M. truncatula*: Choice of substrates and growth conditions. In: The *Medicago truncatula* Handbook. Oklahoma: The Samuel Roberts Noble Foundation; 2006. p. 1–26. https://www.noble.org/medicago-handbook/.

[69] Kurdyukov S, Song Y, Sheahan MB, Rose RJ (2014). Transcriptional regulation of early embryo development in the model legume *Medicago truncatula*. Plant Cell Rep.

[70] Higo K, Ugawa Y, Iwamoto M, Korenaga T (1999). Plant cis-acting regulatory DNA elements (PLACE) database: 1999. Nucleic Acids Res.

[71] Lescot M, Déhais P, Thijs G, Marchal K, Moreau Y, Van de Peer Y, Rouzé P, Rombauts S (2002). PlantCARE, a database of plant *cis*-acting regulatory elements and a portal to tools for *in silico* analysis of promoter sequences. Nucleic Acids Res.

[72] National Centre for Biotechnology Information (NCBI). Primer-BLAST http://www.ncbi.nlm.nih.gov/tools/primer-blast/

[73] Plant System Biology. Gateway™ vectors for functional studies. http://gateway.psb.ugent.be/

[74] Karimi M, Inzé D, Depicker A (2002). GATEWAY vectors for *Agrobacterium*-mediated plant transformation. Trends Plant Sci.

[75] Clough SJ, Bent AF (1998). Floral dip: a simplified method for *Agrobacterium*-mediated transformation of *Arabidopsis thaliana*. Plant J.

[76] Kakar K, Wandrey M, Czechowski T, Gaertner T, Scheible WR, Stitt M, Torres-Jerez I, Xiao Y, Redman JC, Wu HC, Cheung F, Town CD, Udvardi MK (2008). A community resource for high-throughput quantitative RT-PCR analysis of transcription factor gene expression in *Medicago truncatula*. Plant Methods.

[77] Joint Genome Institute (JGI). The plant genomic resource. Phytozome http://phytozome.jgi.doe.gov/pz/portal.html

