# Supplementary material for: Three members of Medicago truncatula ST family are ubiquitous during development and modulated by nutritional status (MtST1) and dehydration (MtST2 and MtST3)
Source: BMC Plant Biol. 2017 Jul 10;17:117. doi: 10.1186/s12870-017-1061-z (PMC5504553; doi:10.1186/s12870-017-1061-z)
Supplement: Supplementary file 1 — Classification of cis-acting regulatory elements found in ST1, ST2 and ST3 promoters under different categories and subcategories. (DOCX 29 kb) [file 12870_2017_1061_MOESM1_ESM.docx]

| **Additional file 1. Classification of *cis*-acting regulatory elements (CRE) found in *ST1, ST2* and *ST3* promoters under different categories and subcategories.** The number of motifs found in each subcategory per promoter is indicated. Motifs are named as listed in the corresponding database. Additional information is available in Additional file 2. | | | | |
| --- | --- | --- | --- | --- |
|  |  |  |  |  |
|  | **nº of CRE per promoter** | | |  |
|  | ***pST1*** | ***pST2*** | ***pST3*** | **MOTIFS** |
| **TISSUE/ORGAN SPECIFIC** |  |  |  |  |
| Meristem | 3 | 1 | 3 | CAT-box, CCGTCC-box, NTBBF1ARROLB |
| Xylem |  | 2 |  | AC-II, XYLAT |
| Root | 7 | 20 | 12 | RAV1AAT, RHERPATEXPA7, ROOTMOTIFTAPOX1, SP8BFIBSP8BIB |
| Mesophyll | 22 | 15 | 15 | CACTFTPPCA1 |
| Guard cell | 4 |  | 6 | TAAAGSTKST1 |
| Flower | 14 | 13 | 16 | GTGANTG10, MYBPLANT, POLLEN1LELAT52, POLLEN2LELAT52 |
| Fruit | 1 |  |  | TGTCACACMCUCUMISIN |
| Monocot seed | 23 | 19 | 27 | -300ELEMENT, ACGTOSGLUB, AMYBOX1, CAREOSREP1, CGACGOSAMY3, DOFCOREZM, DRE2COREZMRAB17, EMHVCHORD, GCN4-motif, MYBGAHV, O2-site, PYRIMIDINEBOXOSRAMY1A, WBOXHVISO1 |
| Dicot seed | 21 | 29 | 23 | CAATBOX1, CARGCW8GAT, DPBFCOREDCDC3, EBOXNNAPA, NAPINMOTIFBN, SEF1MOTIF, SEF4MOTIFGM7S |
| **LIGHT** |  |  |  |  |
| Light | 24 | 48 | 39 | -10PEHVPSBD, A-BOX, ABRELATERD1, ACE, AE-box, AT-1-motif, Box 4, Box I, CACGTGMOTIF, chs-CMA2a, GA-motif, GATABOX, GT1CONSENSUS, IBOX, MNF1, PALBOXAPC, SBOXATRBCS, SORLIP1AT, SORLREP3AT, Sp1, TBOXATGAPB, TCT-motif |
| Circadian |  |  | 3 | CIACADIANLELHC |
| Phytochrome | 3 | 2 |  | REALPHALGLHCB21, REBETALGLHCB21 |
| **HORMONE** |  |  |  |  |
| Citokinin | 5 | 17 | 11 | ARR1AT, CPBCSPOR |
| Abscisic acid |  | 3 | 4 | ABRERATCAL |
| Auxin | 1 |  |  | TGA-element |
| Giberellin | 2 |  | 5 | GAREAT, GARE motif, TATC-box |
| Etilene | 1 | 1 |  | ERELEE4 |
| Salycilic acid | 1 | 1 |  | TCA-element |
| Jasmonate |  |  | 2 | T/GBOXATPIN2 |
| **BIOTIC INTERACTIONS** |  |  |  |  |
| Symbiosis | 3 | 9 | 8 | OSE1ROOTNODULE, OSE2ROOTNODULE |
| Pathogenesis | 12 | 11 | 16 | BIHD1OS, GT1GMSCAM4, LECPLEACS2, MYB1LEPR, SEBFCONSSTPR10A, WBOXATNPR1, WBOXNTCHN48, WBOXNTERF3, WRKY71OS |
| **ABIOTIC STRESS** |  |  |  |  |
| Anoxia | 2 | 5 |  | ANAEROCONSENSUS, ARE, GC-motif |
| Dehydration | 10 | 15 | 17 | CBFHV, MYB1AT, MYB2AT, MYB2CONSENSUSAT, MYBATRD22, MYBCORE, MYBST1, MYCCONSENSUSAT, PREATPRODH |
| Heat shock |  | 1 | 1 | HSE |
| Low temperature | 1 |  | 2 | LTREATLT178, LTRECOREATCOR15 |
| Nutrition | 4 | 5 | 7 | AMMORESIIUDCRNIA1, CURECORECR, EECCRCAH1, IRO2OS, P1BS, SREATMSD, SURECOREATSULTR11 |
| **ENHANCER** |  |  |  |  |
| General |  | 1 | 2 | 5UTR Py-rich stretch, TA-rich |
| Tissue specific | 1 | 3 | 2 | CCAATBOX1, QARBNEXTA, Skn-1 motif |
| **OTHERS** |  |  |  |  |
| Cell cycle |  | 2 | 1 | E2FCONSENSUS, MYBCCOREATCYCB1 |
| Plastid | 1 | 2 | 1 | BOXIINTPATPB, S1BOXSORPS1L21 |
|  |  |  |  |  |
|  | **166** | **225** | **223** |  |
