# Supplementary material for: Three members of Medicago truncatula ST family are ubiquitous during development and modulated by nutritional status (MtST1) and dehydration (MtST2 and MtST3)
Source: BMC Plant Biol. 2017 Jul 10;17:117. doi: 10.1186/s12870-017-1061-z (PMC5504553; doi:10.1186/s12870-017-1061-z)

**Additional file 3. *Medicago truncatula* plants along its development**. Dark-grown seedlings (etiolated) of 24 h post-imbibition (hpi) and 3-, 6- and 10-d-old; Light-grown seedlings (green) of 3-, 6- and 10-d-old; Leaves of stage 1 (small, closed folioles without spot), stage 2 (fully expanded leaves, folioles with spot smaller than 1 cm) and stage 3 (fully expanded leaves, folioles with spot bigger than 1 cm); flowers of stage 1 (green immature with sepals covering the organs), stage 2 (closed yellow flower two days before anthesis), and stage 3 (at anthesis) where the 2 latter stages correspond to stages 0 and 1, respectively, as established by Kurdyukov et al. [69]; pods of stage 1 (early pod with a complete spiral), stage 2 (pod with 5 complete spirals and initial spines with embryo at globular state), and stage 3 (pod with 6 complete spirals and mature spines with embryo at heart state), corresponding to stages 2, 4 and 6, respectively, as established by Kurdyukov et al. [69]; green seeds are seeds collected from 24 to 26 d after pollination at the end of the seed filling phase as established by Verdier et al. [47] when water content has decreased below 50% and desiccation tolerance is increasing exponentially [47]. Bars = 1cm

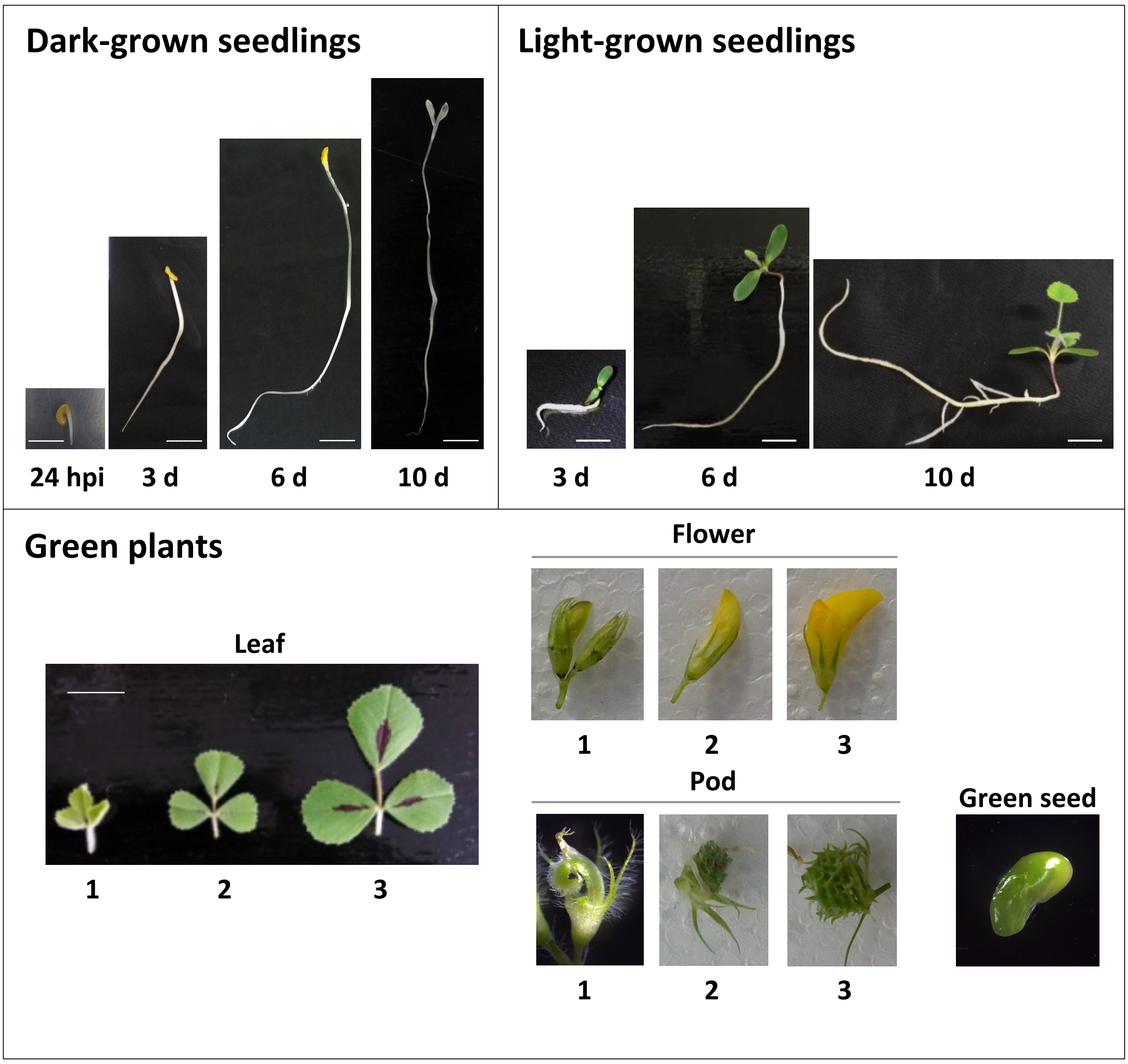

Supplement: Supplementary file 3 — Medicago truncatula plants along its development. (PDF 3399 kb) [file 12870_2017_1061_MOESM3_ESM.pdf]
