# Supplementary material for: Three members of Medicago truncatula ST family are ubiquitous during development and modulated by nutritional status (MtST1) and dehydration (MtST2 and MtST3)
Source: BMC Plant Biol. 2017 Jul 10;17:117. doi: 10.1186/s12870-017-1061-z (PMC5504553; doi:10.1186/s12870-017-1061-z)

Additional file 4. Agarose (2%) gel electrophoresis of sqRT-PCR products. a) analysis of *Medicago truncatula* plants along development: 24 h post-imbibition (hpi) and the root (R) and the aerial (A) parts of 3-, 6- and 10-d-old dark- and light-grown seedlings; and the roots and the leaves of stages 1, 2 and 3; the flowers of stages 1, 2 and 3; the pods of stages 1, 2 and 3 (as established by Kurdyukov et al. [69] and showed in Supplementary figure 1); and the green seeds. **b)** analysis of *Medicago truncatula* 6-d-old seedlings treated during the last 24 h with indolacetic acid (IAA); benzylaminopurine (CK); giberellic acid (GA); epibrassinolide (BL); strigolactone GR24 (SL); abscisic acid (ABA); sodium chloride (NaCl); mannitol; ethephon (ET); salicylic acid (SA); methyl jasmonate (MeJA); as well as a mixture of MeJA with either: ET, GA or SA. Temperature treatment was done for 12 h in darkness in cameras at 4, 25 and 37°C, with 25°C as the control temperature. Finally, for N (-N) and Pi (-Pi) starvations, plants where kept for 7 days either in Fahräeus without N supplement or Fahräeus-N media without phosphate, respectively. Wounding was performed on one foliole by transversally cutting the middle vein. The dotted line inserted between ST3 and UBI results in all gel images indicates that it has been cropped from the original gel and added as a reference.

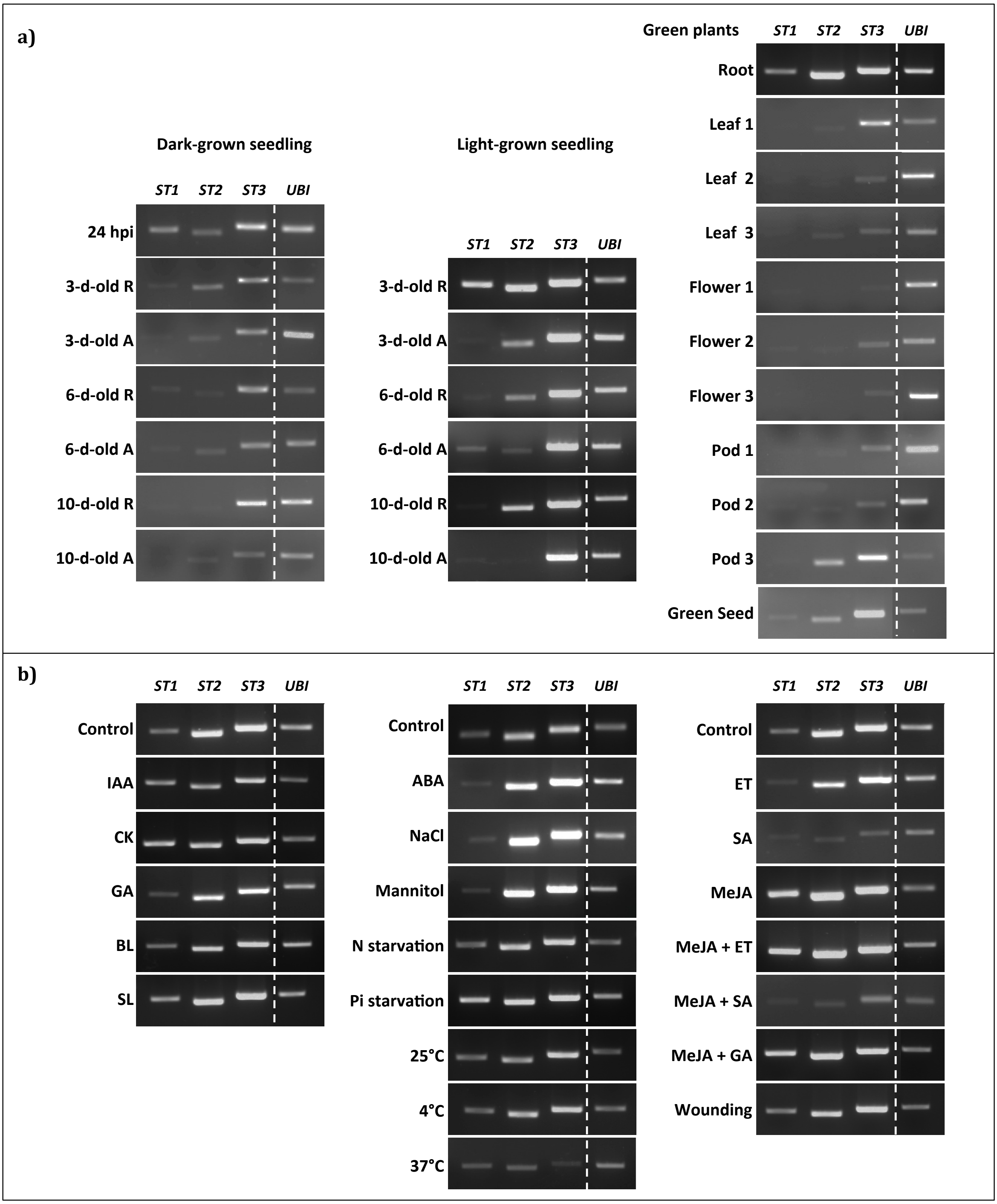

Supplement: Supplementary file 4 — Agarose (2%) gel electrophoresis of sqRT-PCR products. (PDF 850 kb) [file 12870_2017_1061_MOESM4_ESM.pdf]
