# Supplementary material for: Three members of Medicago truncatula ST family are ubiquitous during development and modulated by nutritional status (MtST1) and dehydration (MtST2 and MtST3)
Source: BMC Plant Biol. 2017 Jul 10;17:117. doi: 10.1186/s12870-017-1061-z (PMC5504553; doi:10.1186/s12870-017-1061-z)
Supplement: Supplementary file 5 — Primers used in PCR amplifications. (DOCX 78 kb) [file 12870_2017_1061_MOESM5_ESM.docx]

**Additional file 5.** **Primers used in PCR amplifications.** Tm: melting temperature calculated after 4(G+C)+2(A+T). F, forward primer*;* R, reverse primer*;* Gate, primer used for *Gateway*® cloning (*att*B sequences were added). 3UTR, primer that hybridize in 3’ UTR region. mST, primer used for ORF cloning of corresponding *M. truncatula* gene. pST, primer used for promoter cloning of corresponding *M. truncatula* gene.

| **#** | **Primer sequence (5’→3’)** | **Tm** | **Primer name** |
| --- | --- | --- | --- |
| **Primers used for promoter cloning** | | | |
| 1 | TTCTCAAGTGGGTCATCAATAAA | 62 | pST1.Gate.F |
| 2 | TTTCAAATGTAAAAGCCTCGTTC | 62 | pST1.Gate.R |
| 3 | AATGGGGCGGTTATAAAGATG | 60 | pST2.Gate.F |
| 4 | CTTTGATATTAAAAGCCACTGATTTTATATTTCGATGG | 59 | pST2.Gate.R |
| 5 | CAAGCTTTTGATTTCCAATGTTC | 60 | pST3·F1.Gate.F |
| 6 | TTGCTTTAAAAATTGTTTCTCCAA | 60 | pST3·F2.Gate.F |
| 7 | CTTCTTAAGAATCAGCAAAAGCC | 64 | pST3.Gate.R |
| **Primers used for ORF cloning** | | | |
| 8 | ATGAGACCTGCTCTTGCTGTG | 60 | mST1.Gate.F |
| 9 | CGTCATTATACTTTGTGGCACTTGGC | 64 | mST1.ns.Gate.R |
| 10 | AGAACTTACAGATGGACCACACAT | 60 | mST1.3UTR.Gate.R |
| 11 | ATGAGACCTGCTCTTGCTTTAT | 58 | mST2.Gate.F |
| 12 | CTGCATCATATTTAGTGGCACTTG | 59 | mST2.ns.Gate.R |
| 13 | ATGAGATCTGCTCTTGCTTTAT | 56 | mST3.Gate.F |
| 14 | CAGCTGAAATAATGGGTCTTT | 52 | mST3.ns.Gate.R |
| **Primers used to check the cloning process into Gateway vectors** | | | |
| 15 | TCGCGTTAACGCTAGCATGGATCTC | 64 | L1.F |
| 16 | GTAACATCAGAGATTTTGAGACA | 55 | L2.R |
| 17 | CATCGGCGGGGGTCATAACG | 66 | Kan.F |
| 18 | TGTGGTCGGGGTAGCGGCTG | 66 | eGFP.R |
| **Primers used in sqPCR** | | | |
| 19 | ATGAGACCTGCTCTTGCTGTG | 60 | mST1.sqPCR.F |
| 20 | GTTGCGGGATGCTCATCGC | 62 | mST1.sqPCR.R |
| 21 | CCTGCTCTTGCTTTATTTCCC | 59 | mST2.sqPCR.F |
| 22 | CAAATGGCTCTTCACAATCATG | 58 | mST2.sqPCR.R |
| 23 | CATACAGTACAGAGGCTTTTGC | 60 | mST3.sqPCR.F |
| 24 | CCTTGGTTCAATATCCTCTCC | 59 | mST3.sqPCR.R |
| 25 | GCAGATAGACACGCTGGGA | 59 | mUBI.sqPCR.F |
| 26 | CGCATTCCAGCGGAAGTT | 56 | mUBI.sqPCR.R |
